# A possible unaccounted source of atmospheric sulfate formation: amine-promoted hydrolysis and non-radical oxidation of sulfur dioxide†
†Electronic supplementary information (ESI) available. See DOI: 10.1039/c9sc04756e


**DOI:** 10.1039/c9sc04756e

**Published:** 2020-01-10

**Authors:** Shixian Wang, Xiao Cheng Zeng, Hui Li, Joseph S. Francisco

**Affiliations:** a Beijing Advanced Innovation Center for Soft Matter Science and Engineering , Beijing University of Chemistry Technology , Beijing 10029 , China . Email: hli@mail.buct.edu.cn; b Department of Chemistry , University of Nebraska-Lincoln , Lincoln , Nebraska , USA 68588 . Email: xzeng1@unl.edu; c Department of Earth and Environmental Sciences , University of Pennsylvania , Philadelphia , Pennsylvania , USA 19104 . Email: frjoseph@sas.upenn.edu

## Abstract

Numerous field and laboratory studies have shown that amines, especially dimethylamine (DMA), are crucial to atmospheric particulate nucleation. However, the molecular mechanism by which amines lead to atmospheric particulate formation is still not fully understood. Herein, we show that DMA molecules can also promote the conversion of atmospheric SO_2_ to sulfate. Based on *ab initio* simulations, we find that in the presence of DMA, the originally endothermic and kinetically unfavourable hydrolysis reaction between gaseous SO_2_ and water vapour can become both exothermic and kinetically favourable. The resulting product, bisulfite NH_2_(CH_3_)_2_^+^·HSO_3_^–^, can be readily oxidized by ozone under ambient conditions. Kinetic analysis suggests that the hydrolysis rate of SO_2_ and DMA with water vapour becomes highly competitive with and comparable to the rate of the reaction between SO_2_ and OH·, especially under the conditions of heavily polluted air and high humidity. We also find that the oxidants NO_2_ and N_2_O_5_ (whose role in sulfate formation is still under debate) appear to play a much less significant role than ozone in the aqueous oxidation reaction of SO_2_. The newly identified oxidation mechanism of SO_2_ promoted by both DMA and O_3_ provides another important new source of sulfate formation in the atmosphere.

## Abstract 2

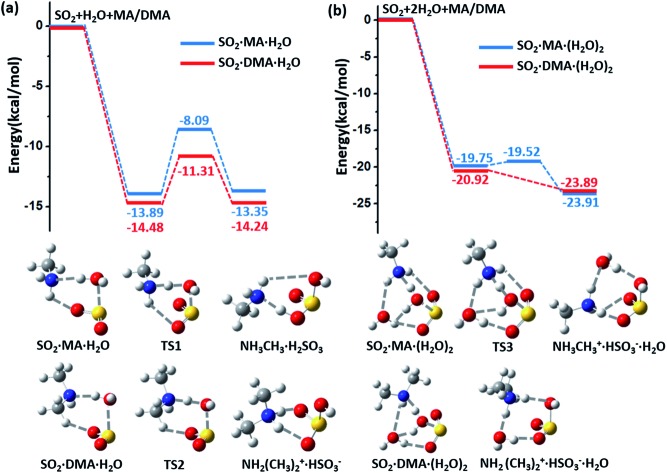
Based on *ab initio* simulations, we show that dimethylamine molecules can also promote the conversion of atmospheric SO_2_ to sulfate.

## 1 Introduction

Sulfuric acid in the atmosphere, mainly produced by the oxidation of gaseous sulfur dioxide, is known as the most important nucleating agent in the earliest stage of atmospheric new particle formation (NPF), as it possesses the lowest vapour pressure (<0.001 mmHg at 298 K) among the gaseous species in the atmosphere.^1^–^9^ SO_2_ in the atmosphere is mainly oxidized by OH· radicals produced from excited oxygen and water vapour.^10^–^12^ However, numerous observations indicate that there is insufficient OH· to account for the unexpectedly rapid growth in H_2_SO_4_ concentration in the highly polluted atmosphere, in which the high aerosol concentration can actually block solar ultraviolet radiation and lower the concentration of OH· radicals, thereby preventing them from participating in photochemical reactions.^5^ Moreover, OH· oxidation alone cannot explain the observed level of H_2_SO_4_ at nighttime.^13^

On the other hand, although the abundance of the common oxidizing gases O_3_ and NO_*x*_ is much higher than that of OH· radicals in the atmosphere, previous *ab initio* calculations show that the direct oxidation of SO_2_ by O_3_/NO_*x*_ in the gas phase is kinetically unfeasible due to extremely high activation barriers. The hydrolysis of gaseous SO_2_ is proposed as an alternative reaction pathway to yield H_2_SO_4_ because sulfurous acid can be more easily oxidized to sulfuric acid by some moderate oxidants, *e.g.*, ozone and NO_*x*_.^14^–^17^ However, the hydrolysis of SO_2_ in the gas phase has also been shown to be both thermodynamically and kinetically unfavourable *via* high-level quantum mechanical (QM) calculations^10^,^18^–^20^ since the hydrolysis of SO_2_ with either H_2_O monomer or dimer is an endothermic process and, again, entails very high energy barriers.^10^ Hence, new oxidation pathways must be explored to explain the fast conversion of SO_2_ to atmospheric H_2_SO_4_.

Atmospheric bases, such as ammonia (NH_3_), are another important contributor to initial sulfate aerosols.^21^ In addition, both cloud chamber studies and field measurements have revealed that atmospheric amines, especially dimethylamine (DMA), also play a surprisingly crucial role in the NPF process, even though their concentrations are two or three orders of magnitude lower than that of NH_3_.^22^–^30^ For example, Almeida *et al.* detected that a 5 pptv level of DMA can enhance the particle formation rate more than 1000-fold than 250 pptv NH_3_.^8^ More recently, Yao *et al.* reported that H_2_SO_4_·DMA·H_2_O nucleation leads to high NPF rates in urban areas of China.^30^ Li *et al.* found that sulfamic acid, produced from SO_3_ and high concentrations of NH_3_, can directly participate in H_2_SO_4_·DMA clustering.^31^ Currently, it is widely accepted that DMA, similar to NH_3_, can further stabilize sulfate clusters through salification with H_2_SO_4_. On the other hand, although Liu *et al.* showed that alkaline gases, such as ammonia, can promote the hydrolysis of SO_2_ to form H_2_SO_3_ (ref. 22) and Chen *et al.* proposed that alkaline aerosols can trap SO_2_, then being oxidized by NO_2_,^14^ the role played by DMA molecules in atmospheric chemistry is still incompletely understood, despite its fundamental importance for exploring the role of amines in atmospheric chemistry.

Here, we show that atmospheric amines can play a key role in the formation of sulfates at high relative humidity (RH) and low illumination, thereby contributing to enhanced aerosol formation on highly polluted days. *Ab initio* simulations demonstrate that the presence of methylamine (MA)/DMA molecules leads to exothermic hydrolysis of SO_2_ with water vapour, without a barrier, to a product that can be oxidized by O_3_ and NO_*x*_. O_3_ is also found to be a stronger oxidant than NO_*x*_ in the amine-assisted oxidation of SO_2_. As a result, the concentration level of atmospheric H_2_SO_4_ from aqueous oxidation may be mainly controlled by the concentration of O_3_ rather than that of NO_*x*_. Based on transition state theory (TST) analysis and the observed concentrations of the participating atmospheric species, the rate of the SO_2_ hydrolysis reaction with the assistance of DMA at 100% RH is even higher than the rate of SO_2_ oxidization by OH·. This finding may shed new light on the long-standing endeavour to identify the unknown oxidation pathway leading to atmospheric sulfate formation.

## 2 Results and discussion

### 2.1 Hydrolysis of SO_2_ assisted by DMA

**Fig. 1:**
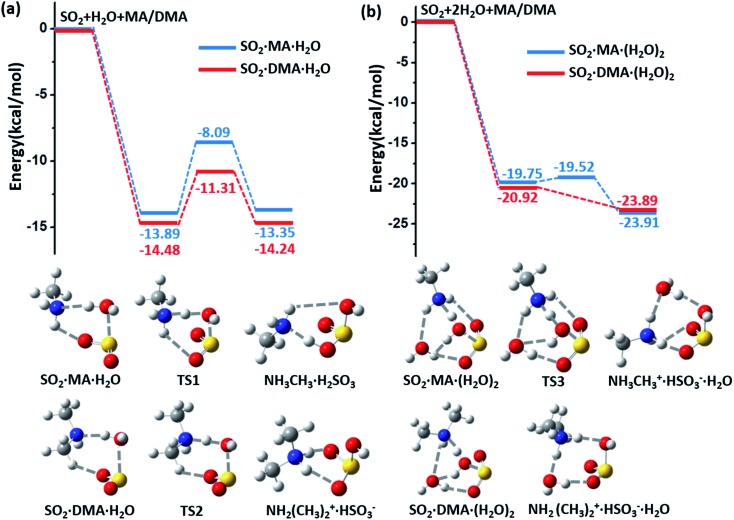
(a) Potential energy profiles for the hydrolysis reactions of MA (blue lines)/DMA (red lines), SO_2_, and H_2_O monomer. (b) Potential energy profiles for the hydrolysis reactions of MA (blue lines)/DMA (red lines), SO_2_, and H_2_O dimer. The energy profiles are calculated at the M06-2X/cc-pVDZ-F12 level with zero-point-energy (ZPE) correction.

The potential energy surfaces (PESs) for the hydrolysis reaction of SO_2_, MA/DMA and *n*H_2_O (*n* = 1–3) are shown in Fig. 1(a–b) and S1 in ESI.† In the reaction with the water monomer (Fig. 1(a)), the breaking of the O–H bond of water in the presence of MA and DMA requires activation energies of 5.8 and 3.2 kcal mol^–1^, respectively, indicating that both reactions can take place quite readily under ambient conditions. In contrast, the process of SO_2_ + H_2_O → H_2_SO_3_ in the gas phase needs to overcome a high energy barrier of 33.9 kcal mol^–1^.^12^ Note that previous quantum-mechanical (QM) calculations showed that atmospheric ammonia can also lower the barrier for the hydrolysis of SO_2_ to ∼12.0 kcal mol^–1^.^22^ However, this energy barrier is still quite high for a reaction to take place at room temperature. According to our calculations, the hydrolysis barriers with MA and DMA are approximately 6.0 and 9.0 kcal mol^–1^ lower than the barrier with NH_3_, respectively, suggesting that amines can promote SO_2_ hydrolysis more strongly than NH_3_. Furthermore, spontaneous ionization to form HSO_3_^–^ and NH_3_CH_3_^+^/NH_2_(CH_3_)_2_^+^ during the hydrolysis reactions is also observed.

The energy barrier for hydrolysis can be further lowered by introducing an additional water molecule to the reaction through the formation of a ring structure in the transition state, as shown in Fig. 1(b). The hydrolysis of SO_2_ with a water dimer and DMA can become barrierless. Likewise, hydrolysis with (H_2_O)_*n*_ (*n* ≥ 3) are also barrierless reactions (ESI Fig. S1†). In addition, increasing the number of water and DMA molecules also promotes further ionization of the already formed H_2_SO_3_ and amine molecules. As shown in ESI Fig. S2†(a), H_2_SO_3_ is partially ionized to HSO_3_^–^ in the (DMA)_2_·H_2_SO_3_·(H_2_O)_*n*_, (*n* = 1–3) clusters, while complete ionization of H_2_SO_3_ and DMA to (NH_2_(CH_3_)_2_^+^)_2_·SO_3_^2–^ is observed in the presence of (H_2_O)_*n*_ (*n* ≥ 4) with a very low dissociation barrier of 0.24 kcal mol^–1^ (ESI Fig. S2(b)†). Next, we show that the complete ionization of H_2_SO_3_ can benefit its oxidation, a phenomenon that may occur on aerosol surfaces (with high pH) in air with high concentrations of DMA and water.

**Fig. 2:**
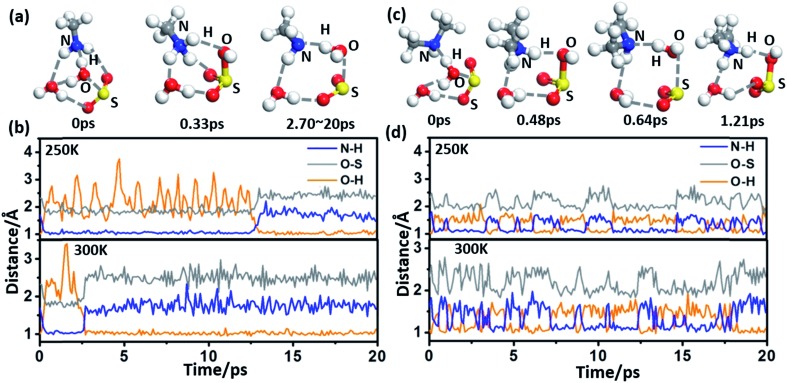
(a) Snapshots taken from the BOMD simulation of SO_2_·MA·(H_2_O)_2_ at 300 K. (b) Time evolution of the O–H, O–S, and N–H bond lengths in SO_2_·MA·(H_2_O)_2_ at 250 and 300 K. (c) Snapshots taken from the BOMD simulation of SO_2_·DMA·(H_2_O)_2_ at 300 K. (d) Time evolution of the O–H, O–S, and N–H bond lengths in SO_2_·DMA·(H_2_O)_2_ at 250 and 300 K, respectively.

The spontaneous formation and ionization processes of bisulfite/sulfite are confirmed by Born–Oppenheimer molecular dynamics (BOMD) simulation (Fig. 2). As shown by the BOMD trajectories at 300 K in Fig. 2(a), the hydrolysis of SO_2_ with a water dimer and MA molecule is a very fast process, where the OH bond of a bridging water molecule breaks during the initial 0.33 ps of the BOMD simulation. Meanwhile, the N–H and O–S bond distances decrease to ∼1.07 and ∼1.78 Å, respectively, suggesting the formation of NH_3_CH_3_^+^·HSO_3_^–^·H_2_O. It is observed that the system does not maintain the ionized form and returns back to the molecular state of SO_2_ after 2.70 ps, indicating the reversible transition between SO_2_ and HSO_3_^–^ due to the thermal effect. It is interesting that such a chemical equilibrium can be sensitively regulated by temperature. The simulation system maintains the form of NH_3_CH_3_^+^·HSO_3_^–^ at 300 K for ∼2.4 ps during the total BOMD simulation time of 20 ps (lower panel in Fig. 2(b)), while the time period that NH_3_CH_3_^+^·HSO_3_^–^ lasts is approximately six times longer at 250 K (∼12.2 ps) than at 300 K (upper panel in Fig. 2(b)). The BOMD simulation of SO_2_·DMA·(H_2_O)_2_ shows a similar trajectory as that of the SO_2_·MA·(H_2_O)_2_ system (Fig. 2(c) and (d)), where the time periods of the NH_2_(CH_3_)_2_^+^·HSO_3_^–^ state are 9.1 and 10.6 ps at 300 and 250 K, respectively. Clearly, lower temperature is beneficial for bisulfite/sulfite formation due to its entropy being lower than that of the loose SO_2_·H_2_O cluster.

Previous studies have suggested that heterogeneous reactions on the surface of water droplets play crucial roles in atmospheric chemistry, such as the ionization of N_2_O_4_.^32^,^33^ Zhong *et al.* found that SO_2_ on a water nanodroplet tends to have an S–O bond exposed to the air that can readily react with other gaseous species in the air.^34^ Here, BOMD simulations also confirm that increasing the size of the water cluster can move the SO_2_ ↔ HSO_3_^–^ equilibrium towards the right-hand side. As shown in ESI Fig. S3(a) and (b),† SO_2_, MA/DMA, and two water molecules quickly convert to NH_3_CH_3_^+^/NH_2_(CH_3_)_2_^+^·HSO_3_^–^·H_2_O cyclic structures, which remain stable on the water cluster during the BOMD simulation at 300 K. By contrast, no similar structure is formed from SO_2_ and NH_3_ during the BOMD simulation (ESI Fig. S3(c)†), implying that the amines have a unique promotion effect on the hydrolysis of SO_2_. The NH_2_(CH_3_)_2_^+^·HSO_3_^–^ complex on the water droplet can also uptake an additional DMA molecule to form (NH_2_(CH_3_)_2_^+^)_2_·SO_3_^2–^, as shown in ESI Fig. S3(d).†

### 2.2 Oxidation of NH_2_(CH_3_)_2_^+^·HSO_3_^–^ and (NH_2_(CH_3_)_2_^+^)_2_·SO_3_^2–^ by O_3_

**Fig. 3:**
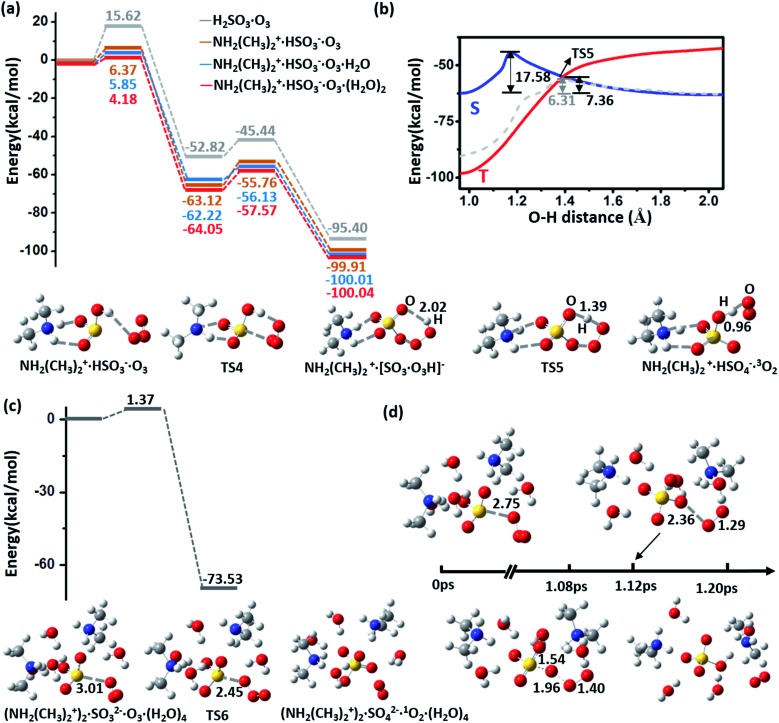
(a) Potential energy profiles for the oxidation reactions of H_2_SO_3_/CH_2_(CH_3_)_2_^+^·HSO_3_^–^·(H_2_O)_*n*_ (*n* = 0, 1, 2) and O_3_. Snapshots are taken from the BOMD simulation. (b) Potential energy *versus* the O–H distance in CH_2_(CH_3_)_2_^+^·[SO_3_·O_3_H]^–^. The blue and red lines correspond to the singlet and triplet multiplicities, respectively. The grey line is obtained from the spin-polarized calculation with the PBE functional in the VASP. Snapshots are taken from the BOMD simulation. (c) Potential energy profiles for the oxidation reactions of (CH_2_(CH_3_)_2_^+^)_2_·SO_3_^2–^·(H_2_O)_4_ and O_3_. Snapshots are taken from the BOMD simulation. (d) Snapshots are taken from the BOMD simulation of (CH_2_(CH_3_)_2_^+^)_2_·SO_3_^2–^·O_3_·(H_2_O)_4_ at 300 K. All energy profiles are calculated at the M06-2X/cc-pVDZ-F12 level with ZPE correction.

The oxidization process of NH_2_(CH_3_)_2_^+^·HSO_3_^–^ by O_3_ is divided into two steps: (1) Adsorption of O_3_ and (2) dissociation of [SO_3_·O_3_H]^–^, as shown in the energy profiles in Fig. 3(a) and (b). The oxidation starts from the physical adsorption of O_3_ with one oxygen atom approaching the HSO_3_^–^ group (*E*_ads_ = –3.03 kcal mol^–1^), and then the O_3_ molecule is chemically adsorbed to HSO_3_^–^ by forming a cyclic structure, NH_2_(CH_3_)_2_^+^· [SO_3_·O_3_H]^–^, as an intermediate state. This process is highly exothermic (Δ*E* = –63.12 kcal mol^–1^) and overcomes a low barrier of 6.37 kcal mol^–1^, which is 9.25 kcal mol^–1^ lower than the energy barrier without an amine (Fig. 3(a)). This energy barrier can be further lowered by adsorption of additional water molecules, where the energy barrier equals 5.85 and 4.18 kcal mol^–1^ for one and two H_2_O molecules, respectively (Fig. 3(a)). Due to the low energy barrier, formation of the NH_2_(CH_3_)_2_^+^·[SO_3_·O_3_H]^–^ complex can spontaneously occur during the BOMD simulation at room temperature (ESI Fig. S4†).

As NH_2_(CH_3_)_2_^+^·[SO_3_·O_3_H]^–^ is an extremely stable intermediate state, the dissociation of [SO_3_·O_3_H]^–^ needs to overcome a relatively high energy barrier (*E*_a_ = 17.58 kcal mol^–1^) to produce HSO_4_^–^ and singlet O_2_ in the spin-restricted calculation (Fig. 3(b)). This barrier seems too high for a room–temperature reaction to occur. However, it is known that unstable singlet O_2_ in the atmosphere can quickly convert to the triplet ground state^35^ through collision and that, in particular, the strong spin–orbit interaction of the heavy element sulfur can greatly enhance the spin-flipping rate. Thus, the real dissociation process is accompanied by a spin-flipping process, which can greatly lower the dissociation barrier. Because the O–H stretching vibration corresponds to the main imaginary frequency of the transition state, we scan the energy surface *versus* the O–H distance (*d*_OH_) of [SO_3_·O_3_H]^–^, as shown in Fig. 3(b). The cross-point (*d*_OH_ = 1.39 Å) between the singlet and triplet Born–Oppenheimer potential surfaces is found to yield a dissociation barrier of 7.36 kcal mol^–1^, indicating the kinetic feasibility of the oxidation process under ambient conditions. The low dissociation barrier (*E*_*a*_ = 6.31 kcal mol^–1^) is also confirmed by a calculation at the spin-polarized Perdew–Burke–Ernzerhof (PBE)/plane-wave level,^36^ as implemented in the Vienna *Ab initio* Simulation Package (VASP 5.3).^37^ The dissociation reaction is also highly exothermic (Δ*E* = –36.79 kcal mol^–1^). Unlike the barrier to the adsorption of O_3_, the dissociation barrier is minimally affected by additional vicinal water molecules (Fig. 3(a) and ESI Fig. S5†).

Moreover, the dissociation of HSO_3_^–^ to SO_3_^2–^, which generally happens on alkaline aerosol surface, can promote oxidation with O_3_. A cluster containing one H_2_SO_3_, two DMA, and four H_2_O molecules is chosen to mimic this situation, where the DMA and H_2_SO_3_ molecules spontaneously form NH_2_(CH_3_)_2_^+^ and SO_3_^2–^. As shown in Fig. 3(c), the oxidation becomes a one-step reaction with an extremely low barrier (*E*_a_ = 1.37 kcal mol^–1^). This oxidation process can be reproduced in the BOMD simulation as well (Fig. 3(d)).

### 2.3 Oxidation of NH_2_(CH_3_)_2_^+^·HSO_3_^–^ with NO_*x*_

**Fig. 4:**
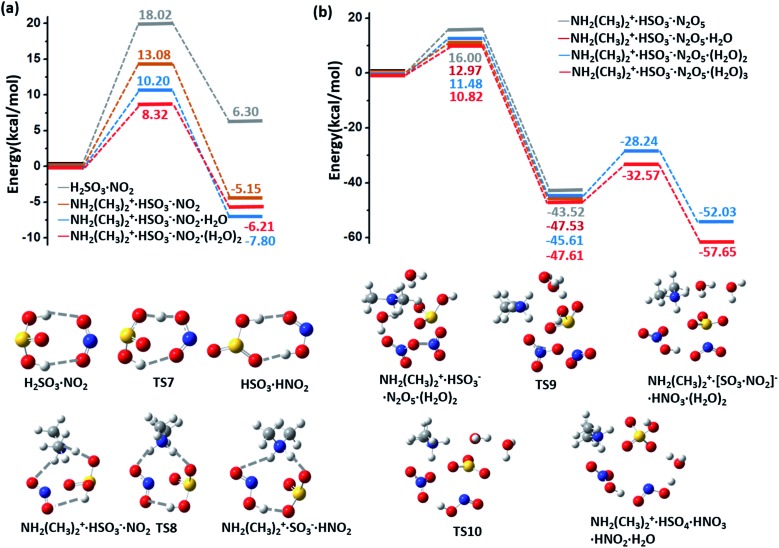
(a) Potential energy profiles for the oxidation reactions of H_2_SO_3_/CH_2_(CH_3_)_2_^+^·HSO_3_^–^·(H_2_O)_*n*_ and NO_2_ (*n* = 0–2). (b) Potential energy profiles for the oxidation reaction for H_2_SO_3_/NH_2_(CH_3_)_2_^+^·HSO_3_^–^·(H_2_O)_*n*_ (*n* = 0,1–3) and N_2_O_5_. The energy profiles are calculated at the M06-2X/cc-pVDZ-F12 level with ZPE correction. Snapshots are taken from the BOMD simulation.

NH_2_(CH_3_)_2_^+^·HSO_3_^–^ can be oxidized by NO_2_ to form the radical NH_2_(CH_3_)_2_^+^·SO_3_^–^ and HNO_2_ (HONO) with an energy barrier of 13.08 kcal mol^–1^ and a potential energy change of –5.15 kcal mol^–1^, as shown by the energy profiles and corresponding structures displayed in ESI Fig. 4(a) and S6(a),† respectively. In contrast to this oxidation reaction, the oxidation process without DMA has a relatively higher barrier (18.02 kcal mol^–1^) and a positive energy change (6.30 kcal mol^–1^), as shown in Fig. 4(a). Similar to the barrier for O_3_ oxidation, the barrier for oxidizing NH_2_(CH_3_)_2_^+^·SO_3_^–^ with NO_2_ can be lowered by extra neighbouring water molecules; *e.g.*, the values of the oxidation barrier in the presence of the water monomer and dimer are equal to 10.20 and 8.32 kcal mol^–1^, respectively. Such a barrier is believed to be even lower on the surface of aqueous aerosols.

The NH_2_(CH_3_)_2_^+^·SO_3_^–^ radical product is an active radical, so it can easily react with other radicals, such as O_2_, NO_2_, and OH˙. For example, our calculations demonstrate that NH_2_(CH_3_)_2_^+^·SO_3_^–^·(H_2_O)_*n*_ (*n* ≥ 1) and another NO_2_ molecule can spontaneously form a NH_2_(CH_3_)_2_^+^·HSO_4_^–^·(H_2_O)_*n*–1_ cluster (ESI Fig. S6(b)†). In addition, HNO_2_, the other product of this oxidation reaction, is also an important precursor of OH˙ radicals in the atmosphere.^38^,^39^

The potential energy profiles of NH_2_(CH_3_)_2_^+^·HSO_3_^–^·(H_2_O)_*n*_ (*n* = 0–3) oxidized by N_2_O_5_, another abundant oxidative NO_*x*_ species in the atmosphere, are shown in Fig. 4(b) and ESI Fig. S6(c).† Similar to O_3_ oxidation, the process of (NH_2_(CH_3_)_2_^+^·HSO_3_^–^·(H_2_O)_*n*_ + N_2_O_5_ → CH_2_(CH_3_)_2_^+^·HSO_4_^–^·(H_2_O)_*n*–1_ + HNO_3_ + HNO_2_) is a two-step reaction, where N_2_O_5_ first dissociates into a NO_2_^–^·NO_3_^+^ ion pair and combines with the bisulfite cluster to form a HNO_3_ molecule and a stable complex [SO_3_·NO_2_]^–^ (Fig. 4(b)). The energy barrier of this step also decreases from 16.0 to 10.82 kcal mol^–1^ as the number of participating water molecule increases (*n* = 0–3). In the second step, a H_2_O molecule that attacks the sulfur atom will assist the breaking of [SO_3_·NO_2_]^–^, and the product CH_2_(CH_3_)_2_^+^·HSO_4_^–^·HNO_3_·HNO_2_·(H_2_O)_*n*–1_ is finally formed. This step is also an exothermic process (Δ*E* = –6.42 and –10.04 kcal mol^–1^ for *n* = 2 and 3, respectively), and the barrier of this step is weakly affected by the number of water molecules (*E*_a_ = 17.37 and 15.04 kcal mol^–1^ for *n* = 2 and 3, respectively). Such high energy barriers indicate that N_2_O_5_ plays a negligible role in the oxidation of sulfite.

### 2.4 Kinetics and implications for atmospheric chemistry

The reaction rate constants of hydrolysis reactions are calculated based on TST, as listed in Table 1, and details of this calculation and the reactant concentrations are listed in ESI Tables S1, S2 and S3.† The rate constant for the hydrolysis reaction of SO_2_·H_2_O with DMA adopts an inverse relation with temperature, decreasing from 3.35 × 10^–10^ to 1.39 × 10^–11^ cm^3^ molecule^–1^ s^–1^ as the temperature changes from 240 to 300 K. According to previous observations, [SO_2_] and [DMA] are ∼10^12^ and ∼10^9^ molecules cm^–3^ in highly polluted air, respectively, while the concentration of H_2_O decreases from 9.7 × 10^17^ to 9.0 × 10^15^ molecules cm^–3^ at 100% relative humility (RH) as the temperature drops from 300 K to 240 K.^7^,^30^,^40^ On the basis of these parameters, the estimated concentrations of the SO_2_·H_2_O and DMA·H_2_O complexes at 300 K are approximately 3.4 × 10^8^ and 1.9 × 10^6^ molecules cm^–3^, respectively, and the rate of hydrolysis for SO_2_ and H_2_O monomer assisted by DMA is estimated to be 4.8 × 10^6^ molecule cm^–3^ s^–1^.

**Table 1:** Values of the total rate constants (*k*, cm^3^ molecule^–1^ s^–1^) for the hydrolysis reactions at temperatures from 240 to 300 K

Reaction	k (cm^3^ molecule^–1^ s^–1^)			
	240 K	260 K	280 K	300 K
SO_2_·H_2_O + MA	6.56 × 10^–13^	3.09 × 10^–13^	1.58 × 10^–13^	8.96 × 10^–14^
SO_2_·H_2_O + DMA	3.35 × 10^–10^	9.42 × 10^–11^	3.21 × 10^–11^	1.39 × 10^–11^
SO_2_·(H_2_O)_2_ + MA	3.79 × 10^–9^	3.17 × 10^–9^	1.18 × 10^–9^	4.22 × 10^–10^
SO_2_·(H_2_O)_2_ + DMA	9.01 × 10^–9^	7.26 × 10^–9^	5.64 × 10^–9^	4.53 × 10^–9^

It is interesting to compare the rate of SO_2_ hydrolysis assisted by DMA to the rate of SO_2_ reacting with OH· radicals under high RH conditions. The latter was previously thought to be the main reaction for SO_2_ oxidation. Using the average concentration of OH· during the daytime (1 × 10^6^ molecules cm^–3^), the reaction rate of the oxidation of SO_2_ by OH˙ based on a previously calculated rate constant (1.3 × 10^–12^ cm^3^ molecule^–1^ s^–1^ at 300 K and 1 atm)^41^ is 1.5 × 10^6^ molecule cm^–3^ s^–1^, which is lower than the hydrolysis rate. In this case, the consumption of SO_2_ in the hydrolysis reaction can exceed that in the oxidation reaction with OH· radicals. Similarly, the estimated hydrolysis rate for atmospheric SO_2_, DMA, and (H_2_O)_2_ at 300 K and 100% RH is 2.9 × 10^6^ molecules per cm^3^ per s, which is also more competitive with the reaction rate of SO_2_ and OH·. Moreover, the concentration of OH· would be further lowered due to the reduced photochemistry either during heavily polluted periods or at night time, when the hydrolysis reaction would even play an even more crucial role in SO_2_ oxidation.

The hydration products CH_2_(CH_3_)_2_^+^·HSO_3_^–^·(H_2_O)_*n*_ are expected to be oxidized by O_3_, NO_2_, and N_2_O_5_. The estimated rate constant of the oxidation of CH_2_(CH_3_)_2_^+^·HSO_3_^–^·(H_2_O)_2_ by O_3_ (5.82 × 10^–15^ cm^3^ molecule^–1^ s^–1^) is 3 orders of magnitude higher than that of the oxidation by NO_2_ (1.73 × 10^–18^ cm^3^ molecule^–1^ s^–1^). As the concentrations of O_3_, NO_2_ and N_2_O_5_ were separately measured to be ∼10^12^, ∼10^12^, and ∼10^10^ molecules cm^–3^ in haze episodes, respectively,^7^,^42^ we can estimate the lifetime of CH_2_(CH_3_)_2_^+^·HSO_3_^–^·(H_2_O)_2_ by the expression *τ* = (*k* × [oxidant])^–1^. The lifetime of CH_2_(CH_3_)_2_^+^·HSO_3_^–^·(H_2_O)_2_ during oxidation by O_3_ is ∼1/1000 of that during oxidation by NO_2_ at 300 K. Considering the much lower oxidation rate constant and concentration of N_2_O_5_ than O_3_ and NO_2_, the oxidation by N_2_O_5_ is negligible. As a result, the proposed hydrolysis of SO_2_ assisted by DMA in an O_3_-polluted atmosphere is an important pathway for sulfate formation.

## 3 Conclusions

**Fig. 5:**
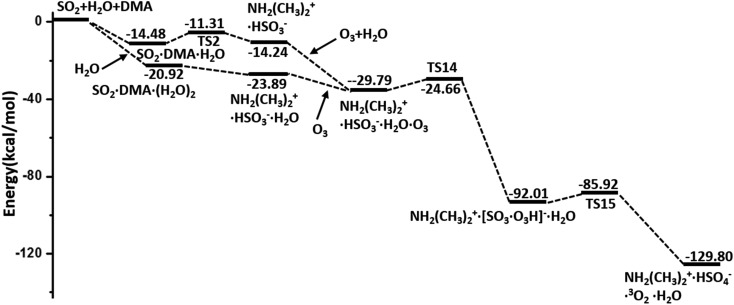
Overall potential energy profiles for the hydrolysis of SO_2_ promoted by DMA and oxidized by O_3_ (M06-2X/cc-pVDZ-F12 with ZPE correction).

In summary, the hydrolysis and oxidation of SO_2_ promoted by DMA are studied by using QM calculations and BOMD simulations. In both gaseous and heterogeneous environments, SO_2_ can be easily hydrated with the assistance of DMA and then oxidized by O_3_, as shown by the overall energy profile in Fig. 5. By contrast, NO_2_ and N_2_O_5_, also viewed as important oxidants in the atmosphere, appear to play a much less important role than O_3_ in the oxidation of SO_2_. Kinetic analysis shows that the consumption rate of SO_2_ during hydrolysis in the presence of DMA can surpass the rate of oxidation with OH· radicals under the conditions of heavily polluted air and high RH.

In the last decade, O_3_ levels in the global atmosphere, according to field measurements, have greatly increased. For example, it has been reported that the yearly mean concentration of O_3_ in Chinese megacities increased by 69% from 2006 to 2015.^43^ The results from this research suggest that the hydrated oxidation of SO_2_ with amines and O_3_ has an important role in atmospheric chemistry.

## 4 Methods

### 4.1 Details of QM calculations and BOMD simulations

The geometries of the reactant states, transition states, and product states in all the reactions are optimized at the unrestricted M06-2X/cc-pVDZ-F_12_ level,^44^–^46^ which has shown good results on weak interactions and has been widely used in computational studies of atmospheric chemistry.^16^,^46^–^48^ Zero-point energy (ZPE) corrections are included when calculating the potential energies, and intrinsic reaction coordinate (IRC) analysis is carried out to confirm the reaction pathways. WB97XD/cc-pVDZ-F12 and B2PLYPD/def2-TZVP methods are also employed for the total potential energy profiles for comparison, which show great consistency with energy profiles based on M06-2X. All the QM calculations for the reaction pathways are performed by using the Gaussian 09 software package.^49^ The spin-polarized calculations are performed based on the generalized gradient approximation of the PBE functional, as implemented in the VASP 5.3.^37^,^50^–^52^ A kinetic energy cutoff of 400 eV is chosen for the plane-wave expansion. The cell size for the NH_2_(CH_3_)_2_^+^·[SO_3_·O_3_H] cluster is 15 × 15 × 15 Å^3^.

BOMD simulations are performed in the framework of the Becke–Lee–Yang–Parr (BLYP) functional^53^,^54^ with the Quickstep module in CP2K code.^55^ The Gaussian and plane wave (GPW) basis sets (280 Ry energy cutoff) combined with the Goedecker-Teter-Hutter (GTH) pseudopotential^56^ are employed to obtain a good balance between computational cost and accuracy. In addition, the dispersion correction is also included to better describe weak intermolecular interactions.^57^ Periodic boundary conditions are used, and the cell sizes for the SO_2_·MA/DMA·(H_2_O)_2_, NH_2_(CH_3_)_2_^+^·HSO_3_^–^·O_3_·(H_2_O)_4_, and (NH_2_(CH_3_)_2_^+^)_2_·SO_3_^2–^·O_3_·(H_2_O)_4_ systems are 20 × 20 × 20 Å^3^. A larger cell size (35 × 35 × 35 Å^3^) is chosen for the hydrolysis reaction of SO_2_ on the surface of a water nanodroplet containing 100 water molecules. The BOMD simulations are carried out at lower and higher temperatures (250 and 300 K), and the temperatures of the systems are controlled using the Nosé–Hoover thermostat. The time step of BOMD is set to 1.0 fs, which has been proven to achieve sufficient energy conservation for water systems.^34^,^47^,^58^ The reaction process is unchanged with a smaller time step of 0.5 fs (ESI Fig. S7†).

### 4.2 Calculation of the reaction rate constant

The rate constants of hydrolysis and oxidation reactions are evaluated using TST with Wigner tunnelling corrections.^48^,^59^,^60^ As [SO_2_][DMA] is negligible relative to [SO_2_][H_2_O] and [DMA][H_2_O], in the hydrolysis reaction of SO_2_ assisted by DMA, two reaction pathways are considered: H_2_O first binding to SO_2_ and first binding to DMA. Because the concentrations of the reactants DMA, SO_2_, SO_2_·H_2_O, and DMA·H_2_O are critical to the final reaction rates, we estimate the number of SO_2_·H_2_O and DMA·H_2_O complexes by the following expressions: [SO_2_·H_2_O] = *K*_SO_2_·H_2_O_[SO_2_][H_2_O] and [DMA·H_2_O] = *K*_DMA·H_2_O_[DMA][H_2_O], where *K*_SO_2__·_H_2_O_ and *K*_DMA·H_2_O_ are the equilibrium constants for the formation of SO_2_·H_2_O and DMA·H_2_O dimers, respectively. The total reaction rate *ν* can be expressed as1
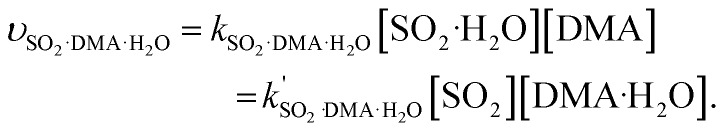



Taking the reaction of SO_2_·H_2_O and DMA as an example, the hydrolysis process is represented by2
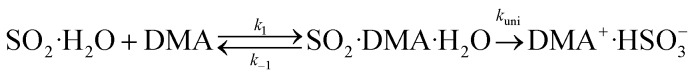



By assuming that the reactant complex SO_2_·DMA·H_2_O is in equilibrium with the reactant monomers SO_2_·H_2_O and DMA, the total rate constant *k*_SO_2_·DMA·H_2_O_ for the reaction can be written as3
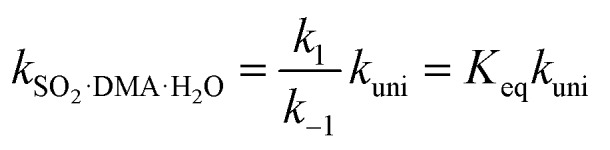
where *K*_eq_ is the equilibrium constant for forming the reactant complex SO_2_·DMA·H_2_O and is expressed by4
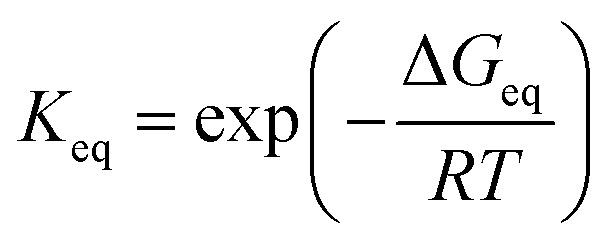
where Δ*G*_eq_ is the free-energy change for the formation of the reactant complex, *R* is the gas constant, and *T* is the temperature. Here, *k*_uni_ is estimated by unimolecular TST and is expressed as

5 *k*_uni_ = *Γk*_2_

The tunnelling effect factor *Γ* is given by Wigner tunnelling corrections,6
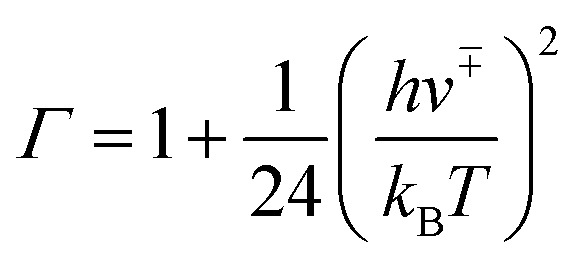
where *h* is the Planck constant, *ν*^+–^ is the imaginary frequency of the transition state, k_*B*_ is the Boltzmann constant, and k_2_ is represented by7
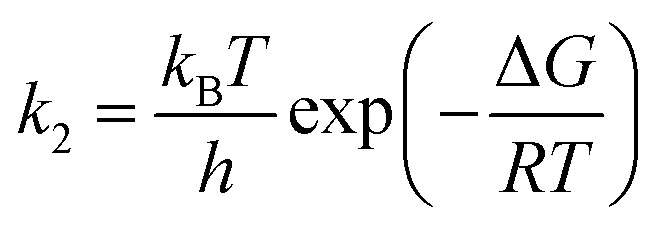
here, Δ*G* is the activation free-energy change from the reactant complex to the transition state. The entropic term *S* is obtained from the partition function *q*(*V*,*T*) as8
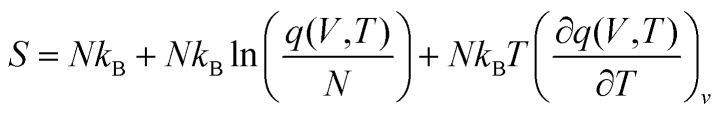
where *q*(*V*,*T*) is determined from the calculation of vibrational frequency.

## Conflicts of interest

There are no conflicts to declare.

## Supplementary Material

Supplementary information
