# Supplementary material for: A possible unaccounted source of atmospheric sulfate formation: amine-promoted hydrolysis and non-radical oxidation of sulfur dioxide
Source: Chem Sci. 2020 Jan 10;11(8):2093–102. doi: 10.1039/c9sc04756e (PMC7059313; doi:10.1039/c9sc04756e)
Supplement: Supplementary file 1 [file SC-011-C9SC04756E-s001.pdf]

Electronic Supplementary Material (ESI) for Chemical Science.
This journal is © The Royal Society of Chemistry 2020

Electronic Supplementary Information

A Possible Unaccounted Source of Atmospheric Sulfate Formation: Amine-Promoted Hydrolysis and Non-radical Oxidation of Sulfur Dioxide

Shixian Wang,[a] Xiao Cheng Zeng,[b,a*] Hui Li,[a*] and Joseph S. Francisco[c*]

[a] Beijing Advanced Innovation Centre for Soft Matter Science and Engineering, Beijing University of Chemical Technology, Beijing, China 100029

[b] Department of Chemistry, University of Nebraska-Lincoln, Lincoln, NE, USA 68588

[c] Department of Earth and Environmental Science and Department of Chemistry, University of Pennsylvania, Philadelphia, PA, USA 19104-6316

Contents

Supplementary Figures……………………………………………………………………………… 2

Supplementary Tables ………………………………………………………………………………7

Cartesian Coordinates of Optimized Structures ………………………………………………….....8

Part 1 Supplementary Figures

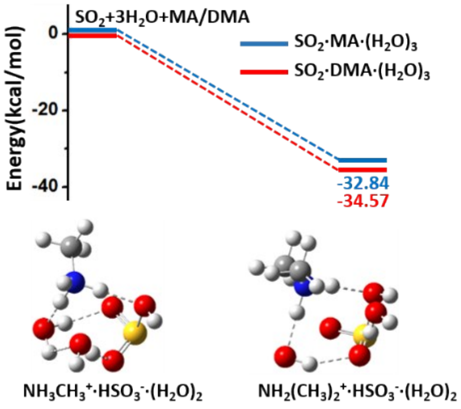

Figure S1 Potential energy profiles for the reaction of MA/DMA and SO_2 with three water molecules. The energy profiles are calculated at M06-2X/cc-pVDZ-F12 level with zero-point-energy (ZPE) correction.

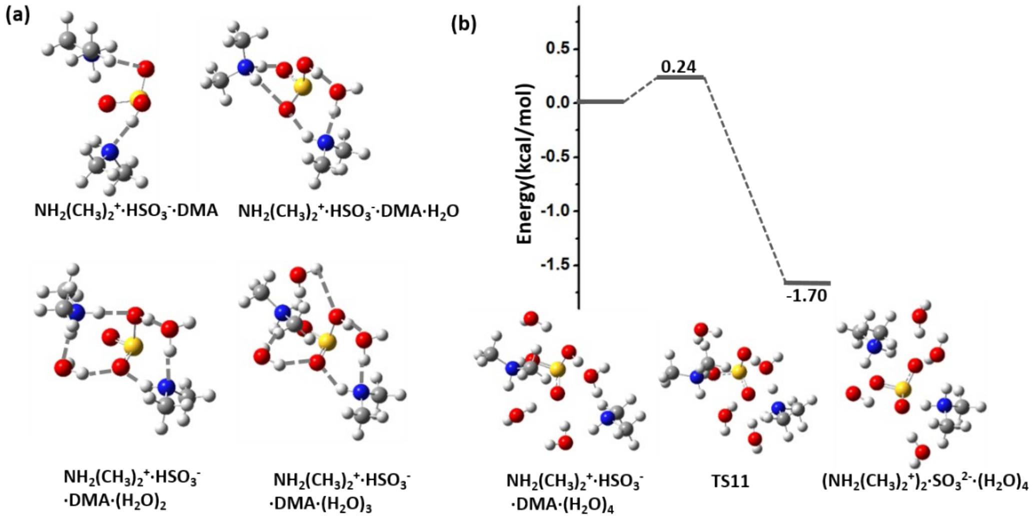

Figure S2 (a) The structures of $NH_2(CH_3)_2^+ \cdot HSO_3^- \cdot DMA \cdot nH_2O \cdot DMA$ (n=0~3). (b) Potential energy profile of the reaction $NH_2(CH_3)_2^+ \cdot HSO_3^- \cdot (H_2O)_4$ and DMA. The energy profile is calculated at M06-2X/cc-pVDZ-F12 level with ZPE correction.

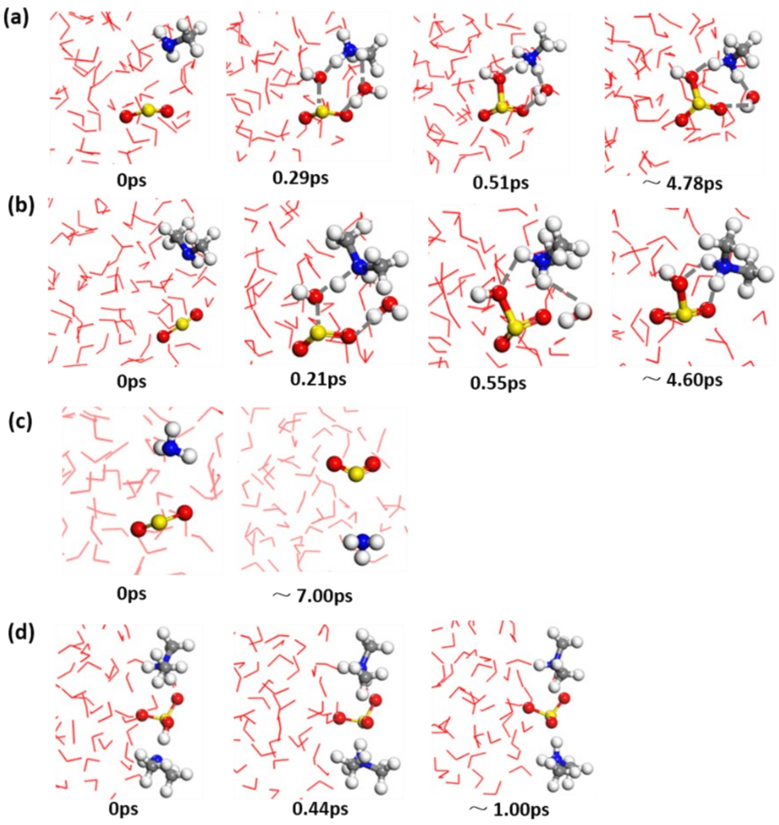

Figure S3 (a) Snapshot structures taken from the BOMD simulation of the SO_2 and MA absorbed on the water surface. (b) Snapshot structures taken from the BOMD simulation of the SO_2 and DMA absorbed on the water surface. (c) Snapshot structures taken from the BOMD simulation of the SO_2 and NH_3 absorbed on the water surface. (d) Snapshot structures taken from the BOMD simulation of the $NH_2(CH_3)_2^+ \cdot HSO_3^-$ and DMA absorbed on the water surface.

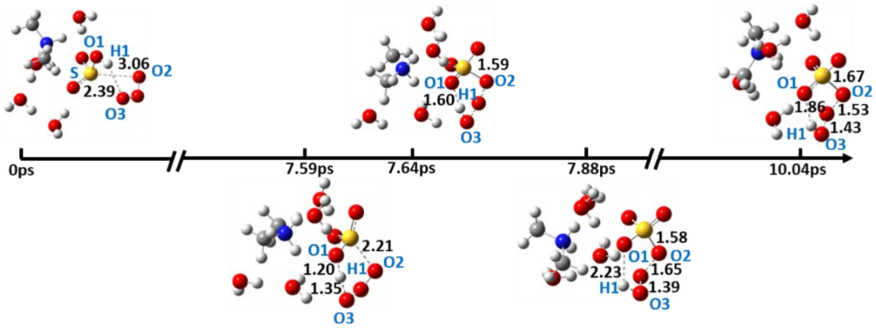

Figure S4 Snapshot structures taken from the BOMD simulation of the system $NH_2(CH_3)_2^+ \cdot HSO_3^- \cdot (H_2O)_4$ and O_3 at 300 K. In the reactant state, four water molecules are H-bonded to the $NH_2(CH_3)_2^+HSO_3^-$ complex. O_2 atom of O_3 molecule interacts with S atom at a distance of 3.06 Å. At 7.59 ps, O1-H1 bond is broken, and S-O2 distance decreases rapidly to 2.21 Å. Thereafter, H1 atom bonds to O3 atom within 0.05 ps, while O2 atom instantaneously bonds to the S atom, forming the stable $NH_2(CH_3)_2^+ \cdot [SO_3 \cdot O_3H]^-$ complex.

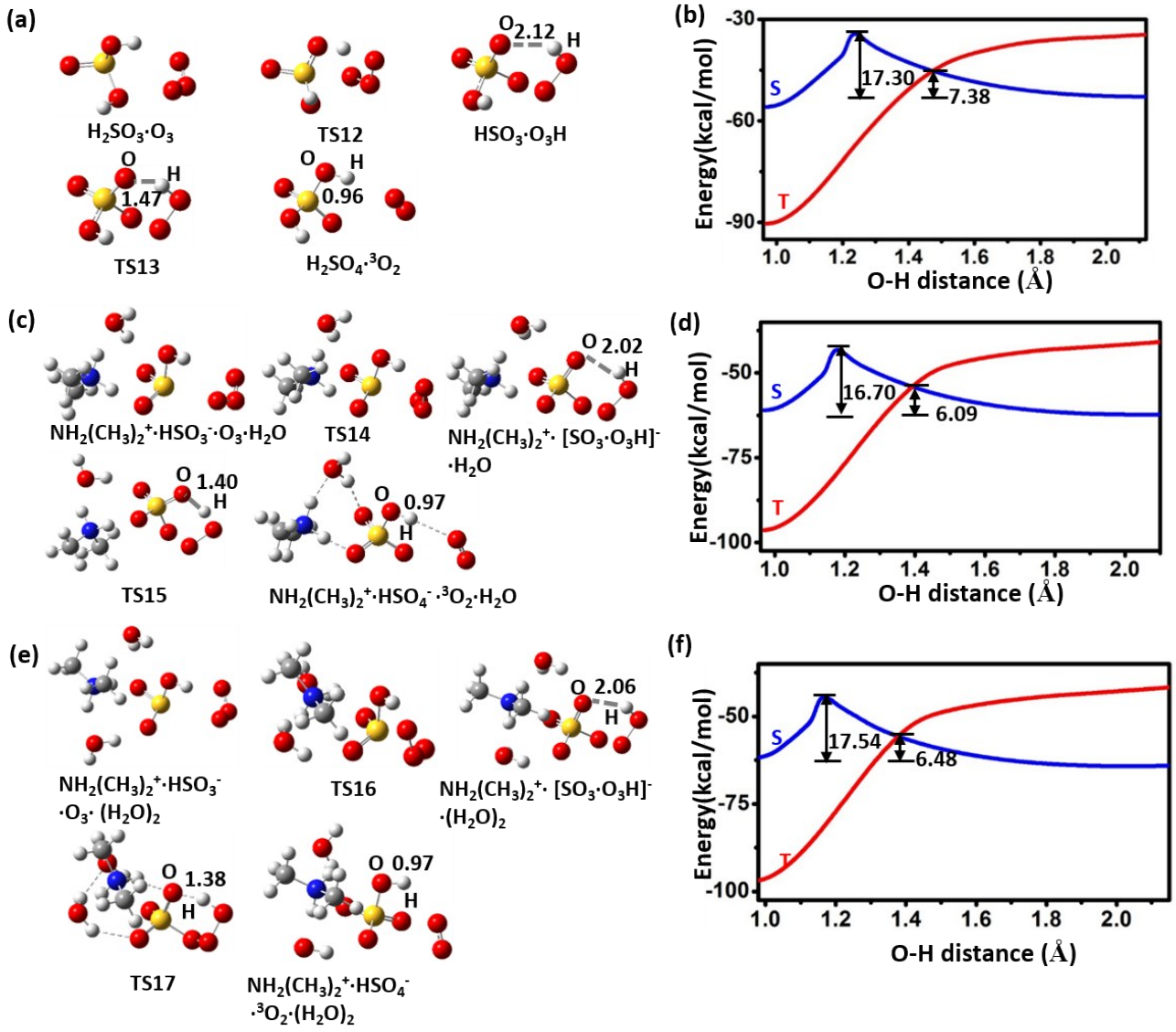

Figure S5 (a) Optimized structures of reactant state, product state and transition state corresponding to the reaction of H_2SO_3 and O_3. (b) Potential Energies versus the O-H distance for $HSO_3{\cdot}O_3H$. (c) Optimized structures of reactant state, product state and transition state corresponding to the reaction of $NH_2(CH_3)_2^+{\cdot}HSO_3^-{\cdot}H_2O$ and O_3. (d) Potential Energies versus the O-H distance for $NH_2(CH_3)_2^+{\cdot}[SO_3{\cdot}O_3H]^-{\cdot}H_2O$. (e) Optimized structures of reactant state, product state and transition state corresponding to the reaction of $NH_2(CH_3)_2^+{\cdot}HSO_3^-{\cdot}(H_2O)_2$ and O_3. (f) Potential Energies versus the O-H distance for $NH_2(CH_3)_2^+{\cdot}[SO_3{\cdot}O_3H]^-{\cdot}(H_2O)_2$.

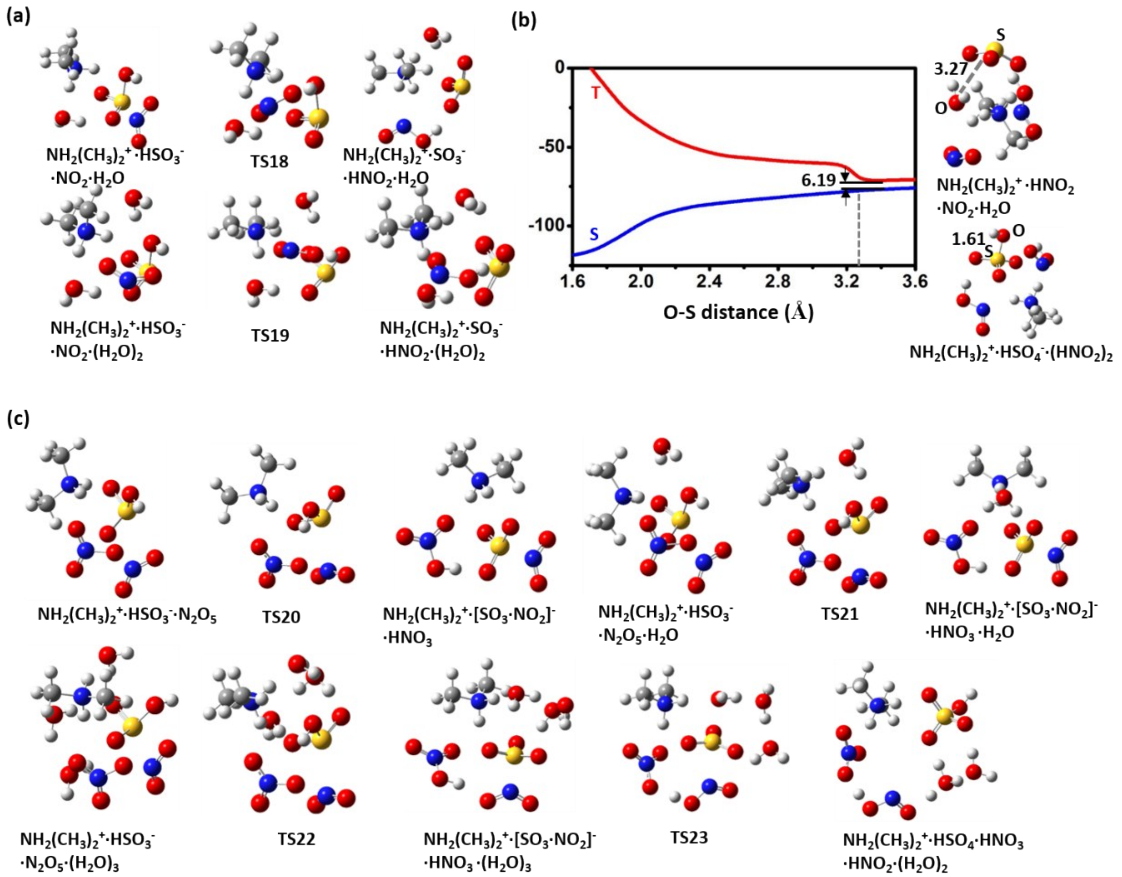

Figure S6 (a) Optimized structures of reactant state, product state and transition state corresponding to the reaction of $NH_2(CH_3)_2^+ \cdot HSO_3^- \cdot (H_2O)n(n=1,2)$ and NO_2. (b) Potential Energies versus the O-S distance for $NH_2(CH_3)_2^+ \cdot SO_3^- \cdot HNO_2 \cdot NO_2 \cdot H_2O$. (c) Optimized structures of reactant state, product state and transition state corresponding to the reaction of $NH_2(CH_3)_2^+ \cdot HSO_3^- \cdot (H_2O)n(n=0,1,3)$ and N_2O_5.

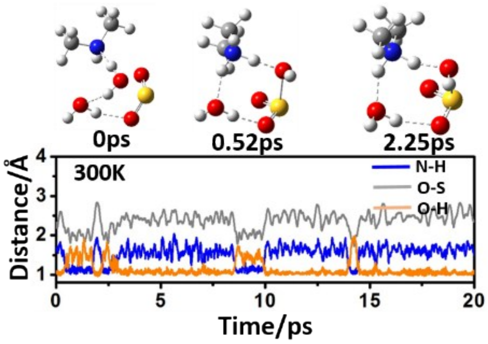

Figure S7 Snapshots taken from the BOMD simulation for $SO_2 \cdot DMA \cdot (H_2O)_2$ with a time step of 0.5 fs; and time evolution of the O-H, O-S, and N-H bond lengths for $SO_2 \cdot DMA \cdot (H_2O)_2$ at 300 K.

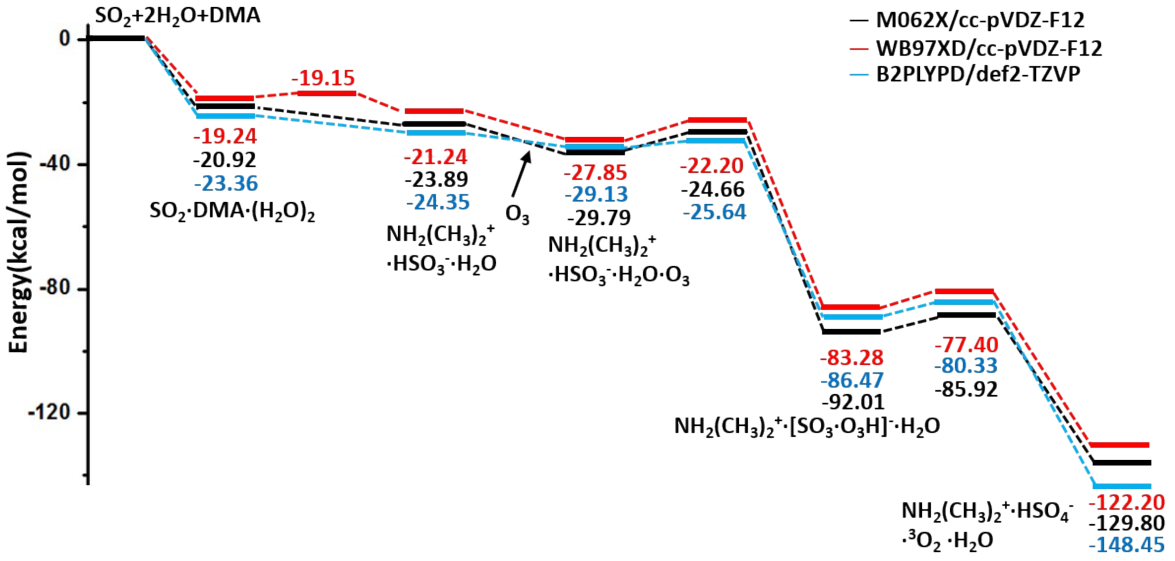

Figure S8 Potential-energy profiles for the hydrolysis of SO_2 promoted by DMA and oxidized by O_3, based on M06-2X/cc-pVDZ-F12 (black lines), WB97XD/cc-pVDZ-F12 (red lines) and B2PLYPD/def2-TZVP (blue lines) methods, respectively.

Part 2 Supplementary Tables

Table S1 Values of the Equilibrium constants (K_{eq}, in $cm^3\cdot molecules^{-1}$) and unimolecular rate constants (k_{uni}, in s^{-1}) for the hydrolysis reactions at temperatures of 240 K-300 K

reaction	T	240K	260K	280K	300K
$SO_2\cdot H_2O$+MA	K_{eq}	4.52×10^{-19}	9.09×10^{-20}	2.33×10^{-20}	7.29×10^{-21}
	k_{uni}	1.45×10^{6}	3.40×10^{6}	6.79×10^{6}	1.23×10^{7}
$SO_2\cdot H_2O$+DMA	K_{eq}	3.93×10^{-19}	7.28×10^{-20}	1.74×10^{-20}	5.58×10^{-21}
	k_{uni}	8.52×10^{8}	1.29×10^{9}	1.84×10^{9}	2.49×10^{9}
$SO_2\cdot(H_2O)_2$+MA	K_{eq}	5.26×10^{-21}	4.58×10^{-21}	1.76×10^{-21}	6.52×10^{-22}
	k_{uni}	7.20×10^{11}	6.93×10^{11}	6.69×10^{11}	6.47×10^{11}
$SO_2\cdot(H_2O)_2$+DMA	K_{eq}	2.23×10^{-21}	2.09×10^{-21}	1.84×10^{-21}	1.66×10^{-21}
	k_{uni}	4.03×10^{12}	3.47×10^{12}	3.06×10^{12}	2.73×10^{12}

Table S2 Values of Free energies (ΔG), Equilibrium constants (K_p), Partial pressures of water monomers ($P(H_2O)$)and dimers($P(H_2O)_2$), and estimated concentration of water monomers ($N(H_2O)$) and dimers ($N(H_2O)_2$) at temperatures of 240K-300K (100% RH)

T	ΔG	K_p	$P(H_2O)$	$P(H_2O)_2$	$N(H_2O)$	$N(H_2O)_2$
240	1.62	0.0331	2.78×10^{-4}	2.56×10^{-9}	9.05×10^{15}	8.33×10^{10}
260	2.06	0.0183	1.99×10^{-3}	7.25×10^{-8}	5.97×10^{16}	2.18×10^{12}
280	2.49	0.0112	1.00×10^{-2}	1.13×10^{-6}	2.79×10^{17}	3.15×10^{13}
300	2.92	0.0074	3.57×10^{-2}	9.42×10^{-6}	9.70×10^{17}	2.65×10^{14}

Table S3 Amount of $SO_2\cdot H_2O$, $DMA\cdot H_2O$, $SO_2\cdot(H_2O)_2$ and $DMA\cdot(H_2O)_2$ in $molecules\cdot cm^{-3}$ at 240 K and 300 K in RH = 100%, with the Equilibrium constants (K_{eq}, in $cm^3\cdot molecules^{-1}$). $[SO_2]= 10^{12}$ $molecules\cdot cm^{-3}$ and $[DMA]= 10^9$ $molecules\cdot cm^{-3}$.

T		$SO_2\cdot H_2O$	$DMA\cdot H_2O$	$SO_2\cdot(H_2O)_2$	$DMA\cdot(H_2O)_2$
240K	K_{eq}	1.71×10^{-21}	2.33×10^{-20}	2.09×10^{-19}	3.11×10^{-18}
	n	1.55×10^{7}	2.11×10^{5}	2.11×10^{4}	3.14×10^{2}
300K	K_{eq}	3.52×10^{-22}	4.00×10^{-21}	1.55×10^{-21}	4.18×10^{-20}
	n	3.42×10^{8}	1.97×10^{6}	6.33×10^{5}	1.71×10^{4}

Part 3 Cartesian coordinates of optimized structures

1. SO_2·MA·H_2O G= -720.859835 a.u.

C	-2.73075800	-0.46449000	-0.34700900
N	-2.01550400	0.12569100	0.79424500
H	-3.35080500	-1.32232200	-0.07832500
H	-1.99669300	-0.78430600	-1.08426900
H	-3.36207000	0.29547500	-0.80447400
H	-1.40119300	-0.57031500	1.20597600
H	-2.66812800	0.41628400	1.51263200
O	-0.15213200	1.64225300	-0.52107900
H	0.18190000	2.39557300	-0.02612100
H	-0.87059300	1.23528500	0.03468200
O	0.47432600	-1.26245200	-0.15525200
S	1.50729000	-0.28192600	-0.34293400
O	2.15830800	0.21422900	0.83247800

2. TS1 G= -720.847549 a.u.

C	-2.55374600	-0.41187300	-0.13467400
N	-1.59015700	0.43934200	0.59630300
H	-3.22209500	-0.92461000	0.55318600
H	-1.97518000	-1.13506600	-0.70257000
H	-3.13023500	0.21243600	-0.81228700
H	-0.99255900	-0.12312800	1.20741700
H	-2.04967100	1.14968300	1.15770500
O	0.21373400	1.14029500	-0.98369000
H	0.63023600	1.98759300	-0.78629400
H	-0.79849800	0.90232900	-0.16074100
O	0.43151600	-1.32736400	-0.34609700
S	1.37167200	-0.22748500	-0.16875800
O	1.36035300	0.30786400	1.18949200

3. $NH_3CH_3^+$·HSO_3^- G= -720.856535a.u.

C	-2.65921400	-0.37835900	0.00634700
N	-1.77847800	0.76068000	-0.30486700
H	-3.12113900	-0.21518200	0.97812200
H	-2.04141200	-1.27251700	0.05728300
H	-3.44120200	-0.52634000	-0.73913100
H	-0.58379000	0.69652000	0.65819600
H	-2.30481600	1.62322200	-0.38457500
O	1.63011800	0.82033000	-0.82387900
H	2.13430000	1.51238300	-0.37522600
H	-1.31068100	0.59815200	-1.19359800
O	0.44980000	-1.25151500	-0.54504600
S	1.25476600	-0.36080200	0.27551800
O	0.29472200	0.46893400	1.20475300

4. SO_2·DMA·H_2O G= -760.130908 a.u.

C	3.13340900	-0.29095500	-0.40713800
N	1.73273600	0.05792000	-0.18689900
C	1.58437400	1.15946100	0.76469900
H	3.73529800	0.56853400	-0.72399500
H	3.20614600	-1.07207200	-1.16177000
H	3.55506600	-0.67442900	0.52268100
H	1.30255300	0.33178800	-1.06515700
H	0.53184300	1.42443800	0.85498400

H	2.15344800	2.04815400	0.47005700
H	1.94537600	0.83456200	1.74187200
O	-0.20262500	-1.73190700	0.52451500
H	-0.22852900	-1.81334700	1.48209300
H	0.59614800	-1.16988300	0.30764100
O	-1.06441900	0.45404100	-1.30044800
S	-1.93488000	-0.07297400	-0.28686700
O	-2.01734500	0.66203600	0.94148200

5.TS2 G=-760.123330 a.u.

C	2.88097200	-0.63124300	-0.10225100
N	1.55739500	-0.00469500	-0.19135600
C	1.54965200	1.42724400	0.15509400
H	3.59328100	-0.13679500	-0.76362100
H	2.80001500	-1.68186200	-0.37108100
H	3.23826800	-0.55470300	0.92331800
H	1.15016700	-0.11527500	-1.12169000
H	0.53053800	1.79785600	0.05868000
H	2.22101700	1.98776100	-0.49573800
H	1.87750300	1.53716500	1.18809900
O	-0.32638700	-1.08321700	1.01396900
H	-0.50379000	-0.73806500	1.89642900
H	0.69772400	-0.56017900	0.49068000
O	-0.96089300	-0.22158500	-1.36530900
S	-1.75708800	-0.25224200	-0.14440700
O	-1.83482300	1.02440500	0.54232500

6. $NH_2(CH_3)_2^+\cdot HSO_3^-$ G= -760.128295 a.u.

C	1.99745400	-1.36245700	0.00604600
N	1.43625600	-0.00564400	0.11759100
C	2.42352200	1.08448000	0.04722900
H	2.51295000	-1.44909600	-0.94813100
H	1.17222300	-2.06951700	0.04284900
H	2.69890700	-1.54771800	0.81791800
H	0.63473600	0.15074900	-0.64028400
H	1.89518100	2.02935200	0.14894600
H	2.92247800	1.04466600	-0.91857200
H	3.15884900	0.97851100	0.84309000
O	-1.36936200	-1.36349600	0.18720000
H	-1.82976200	-1.55707900	1.01351100
H	0.86663500	0.09634200	0.96800900
O	-0.65059200	0.38549100	-1.28256500
S	-1.62901300	0.28618900	-0.14188100
O	-1.04850200	0.90952200	1.07036200

7. $SO_2\cdot MA\cdot(H_2O)_2$ G= -797.279038a.u.

O	-0.75462300	-1.39837500	-0.80616000
S	-1.64748100	-0.76096600	0.12262300
O	-2.15516000	0.52932100	-0.28395900
O	-0.00240700	0.09276200	1.51445100
H	0.82461500	-0.07918400	0.96122300
H	-0.11842500	1.05094300	1.44525300
N	1.99347400	-0.20268100	-0.26795200
C	3.41604800	-0.48121800	-0.04903300
H	1.57733900	-0.88421200	-0.89520700
H	1.85758200	0.70642300	-0.70173800

H	4.01096300	-0.46533900	-0.96515800
H	3.52151900	-1.46103900	0.41331700
H	3.82355400	0.25631500	0.64021800
O	0.06483000	2.30863700	-0.35950800
H	-0.04686100	3.23139700	-0.60108100
H	-0.76231800	1.86745900	-0.60751600

8. TS3 G= -797.276738 a.u.

O	-0.86948700	-0.96076100	-1.18878500
S	-1.62827400	-0.62896400	0.00005500
O	-1.90947900	0.79417000	0.15183300
O	-0.06057300	-0.70277000	1.29962600
H	0.85782600	-0.46797500	0.59747800
H	-0.19556300	0.04136000	1.89950000
N	1.77077800	-0.19667900	-0.31426400
C	3.15919000	-0.54187000	0.02022200
H	1.43493400	-0.68482800	-1.14173600
H	1.64610500	0.80285500	-0.48780400
H	3.84815400	-0.30340900	-0.78932300
H	3.22185100	-1.60553700	0.23854900
H	3.45634600	0.01018500	0.90897900
O	0.41616900	2.33555400	-0.09614400
H	0.28740500	3.24447900	-0.37555900
H	-0.46829700	1.92472000	-0.10468100

9. $NH_3CH_3^+ \cdot HSO_3^- \cdot H_2O$ G= -797.283563 a.u.

O	-0.37419500	-1.14980400	-0.87814000
S	-1.56442100	-0.53211500	-0.20883500
O	-1.61822600	0.92884800	-0.45131100
O	-0.93849000	-0.56949600	1.38084000
H	1.54593700	-0.53128800	1.27281200
H	-1.45189400	0.06658800	1.89421100
N	1.71586600	-0.24761700	0.30962400
C	3.03971700	-0.65556400	-0.19543100
H	0.87757600	-0.68100300	-0.26241900
H	1.55526500	0.77644500	0.25137100
H	3.12266200	-0.34452500	-1.23310200
H	3.12789100	-1.73705700	-0.14065000
H	3.83128800	-0.19134200	0.38729600
O	0.63944500	2.27926100	0.05657700
H	0.74153100	3.03858600	-0.52085800
H	-0.23715900	1.87366800	-0.16580200

10. $SO_2 \cdot DMA \cdot (H_2O)_2$ G= -836.551036 a.u.

O	0.93845900	-1.30698300	0.78616100
S	1.85791100	-0.70906100	-0.14571900
O	2.41398300	0.56221200	0.26310500
O	0.27596100	0.17241200	-1.51087700
H	-0.56736900	0.00790900	-0.94780000
H	0.41544300	1.12661800	-1.43461500
N	-1.77802600	-0.18026800	0.11942500
C	-2.84229400	0.80093700	-0.07709300
H	-1.26030400	0.04848600	0.96358500
H	-2.40895500	1.79920000	-0.07722200
H	-3.61326900	0.74060700	0.69884800
H	-3.31595400	0.62325600	-1.04332400

C	-2.28348000	-1.54871000	0.21078300
H	-3.04467000	-1.66004500	0.99064000
H	-1.45525400	-2.22261300	0.41924200
H	-2.73071200	-1.82584100	-0.74452200
O	0.15115300	2.25615900	0.50204300
H	0.21545100	3.14972600	0.84845900
H	1.00340000	1.83579600	0.69664800

11. $NH_2(CH_3)_2^+\cdot HSO_3^-\cdot H_2O$ G=-836.553763 a.u.

O	0.92576500	-0.32128600	1.29248800
S	1.82076900	-0.34376900	0.12287100
O	1.69926200	0.90682900	-0.67253900
O	0.84733300	-1.41872100	-0.88222300
H	-0.70856500	-0.75152200	-0.38659200
H	1.15902100	-1.29755900	-1.78752400
N	-1.52623900	-0.20159300	-0.04789900
C	-2.48898500	0.00918900	-1.14568600
H	-1.11422000	0.72670000	0.21786800
H	-1.97875700	0.51560900	-1.96105200
H	-3.30778400	0.62940400	-0.78843000
H	-2.87151800	-0.95192300	-1.48204100
C	-2.09714000	-0.86165500	1.14443900
H	-2.89379200	-0.23903800	1.54610500
H	-1.29617800	-0.98098300	1.86815100
H	-2.49722200	-1.83143700	0.85613300
O	-0.42786100	2.29443800	0.18642200
H	-0.19730100	2.59559400	1.07011500
H	0.41844500	1.92131500	-0.16907200

12. $NH_2(CH_3)_2^+\cdot HSO_3^-\cdot O_3$ G=-985.532196 a.u.

C	-2.89982500	1.35016400	-0.25630800
N	-2.73546500	-0.04889500	0.18529800
C	-3.97936100	-0.83106300	0.22773700
H	-3.31542200	1.35090300	-1.26164600
H	-1.91371300	1.81059600	-0.26683900
H	-3.56675000	1.88078300	0.42100100
H	-1.94111200	-0.50458100	-0.44720500
H	-3.75081000	-1.84223700	0.55440800
H	-4.40936800	-0.86249300	-0.77090500
H	-4.68926500	-0.37204200	0.91398500
O	0.54008200	1.16258900	-0.68871300
H	1.33673500	1.55311800	-0.30378900
H	-2.23225000	-0.05517700	1.08246600
O	-0.64812200	-0.89946100	-0.98134800
S	0.39558700	-0.36411700	-0.03172200
O	-0.23422300	-0.10965300	1.28685500
O	3.66649300	-0.32542700	0.27519300
O	3.40483200	-0.34519000	-0.93035900
O	3.09292900	0.52897300	0.97092500

13. TS4 G= -985.517033 a.u.

C	2.54125900	1.12565100	0.85452500
N	2.52481900	0.06160300	-0.17895700
C	3.71750500	0.03000900	-1.04659100
H	3.39437400	0.97693900	1.51228100

H	1.60871700	1.06509900	1.41220300
H	2.61445100	2.08889600	0.35565600
H	2.35451700	-0.83922900	0.28717100
H	3.60098700	-0.76141000	-1.78175500
H	4.60261000	-0.15029100	-0.44070900
H	3.80754800	0.98915700	-1.55041500
O	-0.84465500	0.39845600	1.31692200
H	-1.63986600	0.91478300	0.96598100
H	1.61652900	0.16900400	-0.72342500
O	0.52032900	-1.53103700	0.82961800
S	-0.51073700	-0.69460400	0.20106800
O	0.02058400	0.06661900	-0.95954900
O	-2.83198200	0.36489300	-0.89569700
O	-2.66197200	-0.76479500	-0.35382000
O	-2.82910400	1.37780700	-0.04359500

14. $NH_2(CH_3)_2^+\cdot[SO_3\cdot O_3H]^-$ G= -985.627389 a.u.

C	-3.13845600	1.05912400	0.34369800
N	-2.52119700	-0.27395800	0.15657400
C	-3.46881700	-1.34056100	-0.21768400
H	-3.57416400	1.37492600	-0.60098400
H	-2.35270300	1.74671100	0.64505500
H	-3.90858400	0.99898900	1.10957600
H	-1.72806600	-0.16788200	-0.55927500
H	-2.92253500	-2.27077700	-0.34742100
H	-3.94951800	-1.06561200	-1.15338400
H	-4.22008000	-1.45335900	0.56089100
O	1.55066500	1.44033900	-0.36141500
H	3.35403400	0.53942500	-0.17653600
H	-1.98913300	-0.50329900	1.00194700
O	-0.35818000	0.16065300	-1.23640200
S	0.58360400	0.40362100	-0.13649500
O	-0.09082600	0.43347000	1.14779000
O	2.53125000	-0.87972200	0.68896900
O	1.42229400	-1.01317300	-0.19162800
O	3.60043600	-0.39790800	-0.06581900

15. TS5 G= -985.613484a.u.

C	-2.90244600	1.33409500	0.18943900
N	-2.60140700	-0.11617500	0.19146900
C	-3.76682500	-0.98366200	-0.06184500
H	-3.27571400	1.60843900	-0.79407700
H	-1.97757500	1.86413800	0.40211700
H	-3.65088100	1.55134200	0.94830800
H	-1.80900500	-0.27749100	-0.51346800
H	-3.44528100	-2.02143500	-0.05236400
H	-4.17797200	-0.73766000	-1.03791700
H	-4.52005500	-0.81855300	0.70535500
O	1.36480900	1.21024200	-0.46386400
H	2.50404300	1.01729500	-0.32880500
H	-2.12520600	-0.34531900	1.06994900
O	-0.40044100	-0.28926100	-1.19283800
S	0.57119600	-0.03328100	-0.12263800
O	-0.08212000	0.11933100	1.16309000
O	2.97932400	-0.49491500	0.68620400
O	1.56638800	-1.18507500	-0.12719900

O	3.51753900	0.31497600	-0.13323600

16. $NH_2(CH_3)_2^+ \cdot HSO_4^- \cdot {}^3O_2$ G=-985.633885 a.u.

C	-2.66565400	1.50892900	-0.22683000
N	-2.66518200	0.12037400	0.28229900
C	-3.98918600	-0.52229400	0.30573300
H	-3.02899700	1.50033200	-1.25166700
H	-1.64092800	1.87228700	-0.19956100
H	-3.30876000	2.13116200	0.39243200
H	-1.92757400	-0.43076800	-0.31601300
H	-3.88781700	-1.53214300	0.69391300
H	-4.37579800	-0.56120000	-0.70996900
H	-4.66924500	0.05124700	0.93284600
O	0.99627900	0.80778600	-0.93717800
H	1.96374700	0.74531000	-0.94846100
H	-2.20686600	0.11105300	1.19789700
O	-0.67807000	-0.92655300	-0.93575500
S	0.43366700	-0.43709000	-0.09065200
O	-0.09136100	0.13612700	1.13865400
O	3.72512200	0.53618600	0.87851300
O	1.52980600	-1.35301100	0.04517300
O	3.85933400	0.34243100	-0.28821900

17. $(NH_2(CH_3)_2^+)_2 \cdot SO_3^{2-} \cdot O_3 \cdot (H_2O)_4$ G=-1426.298870 a.u.

O	-0.61464200	-1.99221800	0.62469300
S	0.27602600	-0.94208100	0.05950300
O	0.49939500	0.11395000	1.18831800
O	-0.58315000	-0.13872200	-0.95846500
H	-2.14980100	-0.18992000	-0.14893900
H	0.41289900	2.82798400	-0.22577400
N	-2.98142000	-0.29537400	0.47574900
C	-3.84717000	0.89808900	0.39716900
H	-2.56191500	-0.39002800	1.42991900
H	-3.25900700	1.77409200	0.65595100
H	-4.67864400	0.77766800	1.08847300
H	-4.20833000	0.99209400	-0.62415400
C	-3.66920200	-1.55497200	0.11826700
H	-4.47939600	-1.72790100	0.82372900
H	-2.93359500	-2.35374100	0.16773000
H	-4.05831400	-1.45461100	-0.89209000
O	-1.60223600	-0.64233900	2.87499100
H	-1.35187800	-1.53148900	2.57936200
H	-0.83284700	-0.14631300	2.53386900
N	1.27145400	2.36654900	0.09680000
C	2.07201000	1.97887300	-1.08736600
H	0.94109900	1.44991000	0.57920500
H	1.45250100	1.34965500	-1.72283000
H	2.93290300	1.41634100	-0.73575500
H	2.38340700	2.87480100	-1.62152800
C	2.01879100	3.20196000	1.05911700
H	2.88309300	2.63366200	1.39413400
H	1.37448300	3.43063800	1.90431900

H	2.33224900	4.12392500	0.57249700
O	-1.29096700	2.42991400	-0.99454200
H	-1.84687000	2.49683700	-1.77693000
H	-1.01484600	1.48032500	-0.97675800
O	-2.70729200	0.15839200	-2.57812200
H	-1.84586600	-0.16701100	-2.25268000
H	-2.80736200	-0.18515300	-3.46882800
O	3.26790000	0.31127500	1.46289400
H	2.32158800	0.09689400	1.55672300
H	3.62711100	-0.47523700	1.04054300
O	2.89674100	-2.33171800	-1.20548300
O	3.07642500	-2.43004800	0.02243800
O	2.74209000	-1.18749300	-1.65712100

18. TS6 G=-1426.294762 a.u.

O	-0.67333500	-2.02448300	0.49212500
S	0.21985200	-0.93533600	0.03430700
O	0.46492000	0.02663400	1.21039700
O	-0.56153300	-0.07430600	-0.97516700
H	-2.20278900	-0.12489700	-0.17801100
H	0.59152200	2.78560600	-0.11504500
N	-3.01243200	-0.25147300	0.46059700
C	-3.84555600	0.96858800	0.48221900
H	-2.57700900	-0.42049900	1.39885900
H	-3.22289600	1.81042700	0.77236400
H	-4.65582000	0.82832800	1.19426200
H	-4.23699400	1.13224100	-0.51876800
C	-3.74471700	-1.46682300	0.04066000
H	-4.54157000	-1.66156300	0.75526600
H	-3.03344600	-2.28858100	0.01934800
H	-4.15584500	-1.29136600	-0.95040400
O	-1.66469700	-0.77514400	2.83750100
H	-1.44186400	-1.67584000	2.56064400
H	-0.86686500	-0.30281500	2.52928900
N	1.45949100	2.29743400	0.14192200
C	2.17229800	1.92066800	-1.10346500
H	1.13416200	1.40616500	0.61922600
H	1.49694600	1.32645700	-1.71458700
H	3.03729100	1.32635600	-0.82417200
H	2.46922200	2.82713500	-1.62730700
C	2.28632800	3.10182500	1.06787900
H	3.15157900	2.50492500	1.34316400
H	1.69856400	3.33828800	1.95118700
H	2.58965900	4.01946400	0.56793000
O	-1.18264300	2.51401700	-0.75853600
H	-1.72986700	2.67820800	-1.53287900
H	-0.95096300	1.55626700	-0.82724500
O	-2.71512800	0.41557500	-2.51584300
H	-1.85387700	0.03127400	-2.26899000
H	-2.88279000	0.14388800	-3.42128300
O	3.30243700	0.17897400	1.33139000
H	2.40245600	-0.05397400	1.60401000
H	3.56141400	-0.58526300	0.79366500
O	2.71418800	-2.37333700	-1.18825200
O	3.50547600	-2.29051500	-0.17907000
O	2.10564200	-1.32393200	-1.48214700

19. $(NH_2(CH_3)_2^+)_2 \cdot SO_4^{2-} \cdot {}^1O_2 \cdot (H_2O)_4$ G=-1426.412672a.u.

O	0.66123600	1.75444800	-0.88378800
S	-0.15102800	0.54642800	-0.79208500
O	-0.63006800	0.37118200	0.62870300
O	0.73228000	-0.65599100	-1.07025500
H	2.13174900	-0.09872600	-0.05737500
H	-0.99373300	-2.51454800	0.86248700
N	2.78305200	0.41051400	0.57026300
C	3.44569300	-0.53920000	1.48706900
H	2.17517600	1.07306400	1.10907200
H	2.67884700	-1.08143100	2.03430600
H	4.08202200	0.01770300	2.17141300
H	4.03380500	-1.23470300	0.89338500
C	3.71580600	1.18591300	-0.27591800
H	4.35623500	1.78911500	0.36422300
H	3.11641300	1.81577000	-0.92833200
H	4.31003200	0.48663500	-0.85877600
O	1.04882600	2.08633700	1.96219600
H	0.94515400	2.78299200	1.30105200
H	0.31726400	1.48392100	1.70964700
N	-1.86775300	-2.03965200	0.59253300
C	-2.20230300	-2.43656300	-0.79776500
H	-1.61462100	-1.01563400	0.61177800
H	-1.37891600	-2.14324300	-1.44417600
H	-3.10533200	-1.90939400	-1.09019800
H	-2.35648900	-3.51365900	-0.82447000
C	-2.95351500	-2.31594300	1.55490400
H	-3.82533700	-1.75020800	1.23974200
H	-2.63765000	-1.99582500	2.54440000
H	-3.16473100	-3.38337300	1.55925700
O	0.88063900	-2.62921100	0.70704800
H	1.54717900	-3.21419800	0.33404800
H	0.82278100	-1.88494900	0.05764000
O	3.09548100	-1.87707800	-1.45281900
H	2.25530800	-1.44711700	-1.69421500
H	3.48948700	-2.17919800	-2.27441800
O	-3.60179100	0.24855100	-0.14012300
H	-3.52117900	0.99224100	0.46555800
H	-2.89554100	0.42176800	-0.79394200
O	-2.18453400	3.11903200	-0.51003000
O	-2.04885800	2.78632300	0.62766200
O	-1.30229200	0.52126800	-1.68959900

20. $H_2SO_3 \cdot NO_2$ G= -830.123386 a.u.

O	-0.49427900	0.00005000	1.28535700
S	-1.51299100	0.00020700	0.24448400
O	-1.14408300	-1.23855700	-0.71989400
O	-1.14334900	1.23848800	-0.72029500
H	-0.19768400	1.44674900	-0.62483300
N	2.29861300	-0.00019200	0.11910700
O	1.92280100	-1.09037800	-0.14118000
O	1.92311000	1.09016400	-0.14095500
H	-0.19836500	-1.44685500	-0.62492800

21. TS7 G= -830.091184 a.u.

O	0.79601500	0.23148000	1.37060500
S	1.34228200	-0.26568400	0.11481700
O	1.37692900	0.96774900	-0.89236400
O	0.40632300	-1.26868800	-0.52899800
H	-0.76421000	-1.07608700	-0.28540900
N	-2.21409700	0.31935200	0.04802100
O	-1.37843800	1.16641700	-0.02938800
O	-1.92404300	-0.89825600	-0.06362500
H	0.57208700	1.50194700	-0.73764900

22. $HSO_3 \cdot HNO_2$ G= -830.111602 a.u.

O	-2.76946900	0.16025700	0.28583100
S	-1.44705600	0.09406200	-0.26304900
O	-0.82860400	-1.30517600	0.15063300
O	-0.46205900	1.12597900	0.01946900
H	1.36696800	1.04791400	0.09016400
N	2.80453900	-0.19299800	0.00864100
O	1.95465400	-1.02545900	-0.06467900
O	2.35594500	1.05159600	0.10722200
H	0.15042000	-1.25949300	0.07032400

23. $NH_2(CH_3)_2^+ \cdot HSO_3^- \cdot NO_2$ G= -965.201006 a.u.

C	-1.35705400	-1.17635400	1.33410400
N	-1.66278400	-0.63601800	-0.00537800
C	-2.90638500	-1.14895300	-0.60166500
H	-2.19458700	-0.96010500	1.99382900
H	-0.46603400	-0.66896600	1.70048800
H	-1.19380900	-2.25045200	1.27382100
H	-1.62914600	0.46541900	0.06465800
H	-3.04049700	-0.69667200	-1.58080100
H	-3.74196800	-0.88036900	0.04056700
H	-2.85257400	-2.23203900	-0.69745500
O	0.95670100	1.40754300	1.15460200
H	1.90841300	1.30938900	1.01593900
H	-0.85839800	-0.78868200	-0.62771100
O	-1.15973400	1.86280100	0.11354700
S	0.27893100	1.85762300	-0.32536900
O	0.53488000	0.64884200	-1.15280100
N	1.74952400	-1.38358900	0.05768500
O	2.87396600	-1.03732900	-0.00260700
O	1.11658200	-2.24815700	-0.45501700

24. TS8 G= -965.178503 a.u.

C	2.97104000	-0.05809600	1.10175800
N	1.93029700	-0.13869800	0.05613200
C	2.35951700	-0.84029400	-1.17282300
H	3.25621200	-1.06399100	1.40069600
H	2.56561400	0.48093400	1.95389500
H	3.83487600	0.47052500	0.70551200
H	1.08592300	-0.60941300	0.44439900
H	1.52290300	-0.85019500	-1.86546300
H	2.64602800	-1.85803700	-0.91671400
H	3.20800800	-0.31418200	-1.60433900
O	-2.58540000	-0.32045600	0.47283200
H	-2.36109000	0.63558200	0.32894300
H	1.59088300	0.82909300	-0.17219100

O	-0.40176800	-1.32373900	1.03894300
S	-1.39926800	-1.23783300	-0.04775800
O	-0.85010700	-0.57998900	-1.25180500
N	-0.41482600	1.59681700	-0.01884400
O	-1.48772500	2.16705800	0.04202400
O	0.63091200	2.21569200	-0.22015000

25. $NH_2(CH_3)_2^+ \cdot SO_3^- \cdot HNO_2$ G= -965.208074a.u.

C	-2.54798500	-0.71780700	0.87991800
N	-1.77811000	0.21084700	0.02572600
C	-2.60374600	1.14247200	-0.77438800
H	-3.16718800	-0.13347800	1.55618600
H	-1.85002500	-1.33028100	1.44302100
H	-3.17126700	-1.35072700	0.25303800
H	-1.08687900	0.77016100	0.60134200
H	-1.93098500	1.75707900	-1.36593000
H	-3.18669700	1.76032100	-0.09605500
H	-3.26729200	0.57021800	-1.41881100
O	2.35451800	0.48967300	0.35287300
H	2.10898100	-1.12703500	0.03360600
H	-1.15360300	-0.31122800	-0.59623400
O	0.25603200	1.52549700	1.14235900
S	1.23380800	1.38757800	0.02785900
O	0.55807000	1.07624500	-1.24281700
N	0.41701900	-1.83030000	-0.06851000
O	1.72769200	-2.03438700	-0.13511900
O	-0.22105500	-2.80929100	-0.26599600

26. $NH_2(CH_3)_2^+ \cdot HSO_3^- \cdot N_2O_5 \cdot (H_2O)_2$ G=-1398.298803 a.u.

S	-0.58101800	-1.86068500	0.72350000
O	-1.08037300	-2.40550200	-0.81000900
O	0.09736900	-0.61211600	0.28375500
O	-1.87861200	-1.51460600	1.36996600
H	-1.32149500	-3.33649100	-0.72070400
H	-2.30272300	0.80233900	-0.83100600
N	-1.46763500	1.43229900	-0.81676300
H	-0.93424300	1.09192400	-0.00193900
C	-0.68063200	1.18975400	-2.04502800
H	-1.27761500	1.47642800	-2.90817200
H	0.22988900	1.78239900	-2.00165500
H	-0.42565700	0.13348500	-2.08062600
C	-1.85798500	2.84146500	-0.61401700
H	-0.95589500	3.44741000	-0.56862200
H	-2.47999800	3.16032700	-1.44730200
H	-2.39945800	2.91104900	0.32414800
O	2.42060800	0.73395300	1.68288200
O	2.24456300	-0.92762100	-1.86942000
N	2.51437100	-0.66032100	-0.75434000
N	1.89759400	1.22963800	0.75054000
O	2.28311900	0.80970000	-0.56475600
O	2.95426100	-1.26919900	0.14439200
O	1.10122100	2.11776000	0.68150000
O	-3.35170900	-0.57945000	-0.80377200
H	-2.89264700	-1.23233300	-1.34854600
H	-3.10733400	-0.88672900	0.09216300
O	-1.72246300	1.22157200	2.01980400

H	-0.94533900	1.37488800	2.56423500
H	-1.81366600	0.25171100	1.97551900

27. TS9 G=-1398.275701 a.u.

S	0.31511400	-1.31479500	-0.07039000
O	-0.08134100	-0.65087100	-1.53650400
O	-0.08488800	-0.29848200	0.91906800
O	-0.54841200	-2.50113900	-0.07077000
H	0.53907000	0.08371500	-1.68965400
H	-2.44492300	0.63840800	-0.57717000
N	-2.07384000	1.40226000	0.04339100
H	-1.08057400	1.16629400	0.18782400
C	-2.20917300	2.72174900	-0.60364300
H	-3.26368800	2.92019900	-0.78054200
H	-1.78300100	3.48020500	0.04795200
H	-1.66329600	2.70783900	-1.54238700
C	-2.75914600	1.29169400	1.35175200
H	-2.33408600	2.02229300	2.03584600
H	-3.82012100	1.48370800	1.20749000
H	-2.60621800	0.28351400	1.72829500
O	2.27765800	2.01393000	0.99718300
O	3.36742500	-1.37593800	-0.68471600
N	2.82857300	-0.81600300	0.16766600
N	1.72120200	1.71403600	-0.02652600
O	2.23119100	0.74844400	-0.76661500
O	2.82631400	-0.61555700	1.29760900
O	0.70417200	2.24001900	-0.46524100
O	-3.04143200	-0.73375800	-1.35889000
H	-2.22328500	-1.08639200	-1.73620200
H	-3.24544300	-1.33273600	-0.61500000
O	-3.03532000	-2.10816500	1.03613100
H	-3.45070200	-2.92296200	1.32855200
H	-2.11011600	-2.33793500	0.81282700

28. $NH_2(CH_3)_2^+ \cdot [SO_3 \cdot NO_2]^- \cdot HNO_2 \cdot (H_2O)_2$ G= -1398.373679 a.u

S	1.02185500	-0.82786700	0.19055400
O	0.03435500	-0.32765500	1.11535500
O	1.04134700	-0.18586700	-1.09970400
O	2.29584900	-1.19392400	0.74413900
H	-2.00924000	-1.54984000	0.50000600
H	0.66755100	2.14226200	0.19121600
N	-0.19961400	2.22212300	-0.39290200
H	-0.50738100	1.25837300	-0.55725300
C	-1.24643800	2.93524900	0.37003700
H	-0.91314900	3.95303800	0.55788800
H	-2.17026000	2.93008900	-0.20327300
H	-1.40044800	2.41320100	1.31163900
C	0.13417000	2.83680400	-1.69570000
H	-0.76391900	2.87160900	-2.30791100
H	0.51191100	3.84194400	-1.52453000
H	0.89187600	2.22120100	-2.17300300
O	-3.75856900	0.81953200	0.63934800
O	-0.90003000	-2.67350900	-0.11137300
N	0.30094600	-2.50873400	-0.26927400
N	-2.94485200	0.05425200	0.21055500
O	-2.80563800	-1.08913000	0.88814100

O	1.05325300	-3.31059200	-0.73265500
O	-2.24421100	0.21256000	-0.77358000
O	2.00969500	2.07616700	1.22743600
H	1.81622000	1.44764700	1.93237800
H	2.83444400	1.74604100	0.80956000
O	4.10225000	0.85378000	-0.03385500
H	5.00658100	0.78562100	0.28236100
H	3.71877500	-0.03470900	0.04388600

29. TS10 G= -1398.343290 a.u.

S	1.25731300	0.01912600	-0.35980600
O	0.18217000	-0.13432000	-1.27264600
O	1.10062500	-0.36882700	1.00101500
O	2.43910600	0.70183200	-0.75741000
H	-1.30429200	2.74402100	-0.07297300
H	-0.34974600	-2.04638900	0.86605600
N	-1.34937800	-2.01457500	0.66105000
H	-1.58229400	-0.95396900	0.57508600
C	-1.63735400	-2.73854200	-0.59784400
H	-1.34460500	-3.78111800	-0.48652800
H	-2.70062900	-2.65234800	-0.79907300
H	-1.08430200	-2.26294400	-1.40172500
C	-2.13008000	-2.51042300	1.81386700
H	-3.18278500	-2.34732200	1.59470400
H	-1.93781200	-3.57016200	1.96794200
H	-1.85228400	-1.94032600	2.69635800
O	-3.11646500	-0.15881900	-0.97423700
O	-0.60420600	3.28991500	0.39053100
N	0.47957400	2.51677300	0.44710000
N	-2.51158600	0.75426300	-0.42294700
O	-2.45716800	1.89357300	-0.90064800
O	1.37953200	3.02222400	1.01896700
O	-1.90249600	0.52674800	0.67206300
O	2.08735600	-2.01837400	-0.83791700
H	2.25253100	-2.06784800	-1.78680000
H	2.96860800	-1.92552300	-0.40361200
O	4.34475500	-1.16719100	0.33953200
H	4.53914400	-1.25943500	1.27639900
H	4.11011400	-0.23817200	0.20449400

30. $NH_2(CH_3)_2^+ \cdot HSO_4^- \cdot HNO_3 \cdot HNO_2 \cdot H_2O$ G= -1398.381402 a.u.

S	-0.05576700	-1.76279200	-0.15870200
O	-1.29817500	-2.37684800	0.31733800
O	0.97295800	-1.70073500	0.85467200
O	-0.32050000	-0.53273700	-0.88360000
H	1.18565500	2.70148300	-0.39290800
H	-2.30300200	-1.08435600	0.57533600
N	-2.78042800	-0.14726900	0.51222000
H	-2.00957800	0.52701900	0.43902000
C	-3.54856300	-0.11950100	-0.75350400
H	-4.30651200	-0.89831800	-0.71384600
H	-4.00670300	0.85871000	-0.87073400
H	-2.84829000	-0.30236000	-1.56355700
C	-3.58204900	0.14865300	1.71601700
H	-4.01373200	1.14200700	1.61666400
H	-4.37170700	-0.59311600	1.80724300

H	-2.93436600	0.11146400	2.58746500
O	-1.70408300	2.26742800	-0.95365600
O	2.56236900	2.39064200	0.29383100
N	2.70860800	1.20702800	0.13500600
N	-0.66826300	2.30946600	-0.33840700
O	0.36323100	2.85398500	-0.97322900
O	3.82381600	0.77312300	0.64725900
O	-0.50013900	1.90897300	0.79437800
O	0.57686800	-2.78105100	-1.22936200
H	0.06955000	-2.72140400	-2.04878700
H	3.64474500	-2.02288100	-0.66136000
O	3.56587200	-1.70551100	0.24279700
H	2.61290300	-1.80956900	0.47918000
H	3.79982400	-0.24163800	0.46529200

31. $NH_3CH_3{}^+\cdot HSO_3{}^-\cdot(H_2O)_3$ G= -873.707224 a.u.

O	-0.52336000	-1.47116000	-0.56535700
S	-1.58976800	-0.62840600	0.02047700
O	-1.51753700	0.76051600	-0.51269700
O	0.93721700	0.26633900	-2.14203600
H	0.37573400	-0.49625000	-1.90230200
H	0.29742400	0.98843000	-2.17278800
N	1.70205800	0.11891500	0.49952300
C	2.47846700	-1.13844000	0.57904600
H	1.44824100	0.32181300	-0.49907300
H	1.84479900	-1.92812000	0.18399900
H	2.73025500	-1.34708800	1.61490500
H	3.38174900	-1.04464800	-0.01689600
H	2.17692900	0.93872700	0.87110900
O	0.35405000	2.45292400	0.52024000
H	0.09974500	3.37694200	0.47399400
H	-0.39063200	1.94492800	0.13080100
O	-0.85032800	-0.39113600	1.57537000
H	-1.31376200	0.33909300	2.00617600
H	0.80025900	0.01904200	1.00732900

32. $NH_2(CH_3)_2{}^+\cdot HSO_3{}^-\cdot(H_2O)_3$ G= -912.980192 a.u.

O	-0.94114200	-1.64156600	0.34415800
S	-1.77696500	-0.42690100	0.37164900
O	-1.55658200	0.38116300	-0.87597400
O	-0.83538300	0.49366200	1.49091000
H	0.74927600	0.25836000	0.71543700
H	-1.01847500	1.43520900	1.34439000
N	1.57107500	-0.10379000	0.18841000
C	2.55134500	0.97157100	-0.07042800
H	1.17461900	-0.45677400	-0.71574500
H	2.03974100	1.78836200	-0.57131700
H	3.35236600	0.57523800	-0.69043500
H	2.95564800	1.31762600	0.87833900
C	2.12239300	-1.25806400	0.93269100
H	2.92288800	-1.70554700	0.34744300
H	1.31345300	-1.96675000	1.08898300
H	2.51080100	-0.90960000	1.88702200
O	-0.22575200	2.65282700	-0.29207500
H	-0.53180800	3.47408300	-0.68404900
H	-0.76448500	1.93698100	-0.69082100

O	0.42637000	-1.14148100	-2.12374100
H	0.06769800	-1.90914500	-1.65612600
H	-0.32032700	-0.51897100	-2.03818200

33. $NH_2(CH_3)_2^+ \cdot HSO_3^- \cdot$DMA G= -895.199713 a.u.

O	0.57737400	-0.77937200	-1.25832400
S	-0.08053600	-1.47362500	0.07510900
O	-1.42397600	-1.83968000	-0.43032000
O	-0.22056100	-0.24442800	0.99020100
H	1.76822000	0.97614500	0.67453400
H	-1.45542800	0.37236900	0.61964000
N	2.52415600	0.64954400	0.07880700
C	3.33937400	1.76682800	-0.37920800
H	1.36858700	-0.25643200	-0.94948100
H	2.71232500	2.49941000	-0.88560800
H	4.07780500	1.40139800	-1.09389700
H	3.87587300	2.26459800	0.43805400
C	3.30354500	-0.33637400	0.82267100
H	4.02687000	-0.80132400	0.15162200
H	2.63726000	-1.11026600	1.20321600
H	3.84900400	0.10596700	1.66492400
N	-2.44119800	0.59651100	0.11912400
C	-3.43307700	1.22828300	0.99816500
H	-2.68668400	-0.37091400	-0.13261200
H	-3.56322000	0.61434300	1.88588600
H	-4.38526200	1.33425700	0.48010700
H	-3.07175900	2.21212000	1.29050100
C	-2.15535900	1.33775800	-1.12374900
H	-3.06471400	1.44873800	-1.71249200
H	-1.40611800	0.77420800	-1.67753100
H	-1.76447600	2.31985900	-0.86385200

34. $NH_2(CH_3)_2^+ \cdot HSO_3^- \cdot$DMA$\cdot H_2O$ E= -971.624042 a.u.

C	3.51209300	0.21513000	-1.31958300
N	2.67989300	-0.58970000	-0.41243700
C	3.25256800	-1.88609400	-0.02018600
H	4.45900600	0.42797200	-0.82752400
H	2.98366900	1.14436200	-1.51909100
H	3.69757500	-0.32445100	-2.24755500
H	2.40095000	0.01143700	0.51855400
H	2.54622100	-2.37873200	0.64401500
H	4.18894300	-1.71100200	0.50550000
H	3.43817800	-2.50374900	-0.89808700
O	0.59373600	1.49193100	-0.40751800
H	-0.27552100	1.57952100	-0.87186000
H	1.74493200	-0.73018200	-0.80727200
O	1.73562500	0.70505300	1.53109200
S	0.30674300	0.71782300	1.02585400
O	-0.04164200	-0.66057300	0.57414500
O	-1.88463200	1.57622500	-1.49663500
H	-2.41137200	2.35681300	-1.31012900
H	-2.34748100	0.79981300	-1.05683300
N	-2.88504100	-0.58222600	-0.19633300
H	-1.96022800	-0.97898600	-0.03399400
C	-3.74241800	-1.52584400	-0.90188500
H	-4.68968900	-1.04227600	-1.14659200

H	-3.26697200	-1.83044400	-1.83300500
H	-3.96312700	-2.42099100	-0.30779500
C	-3.43781200	-0.19293200	1.09732800
H	-2.71942600	0.43800500	1.61996400
H	-4.35775600	0.37520300	0.94579300
H	-3.67104900	-1.05664200	1.73093700

35. $NH_2(CH_3)_2^+\cdot HSO_3^-\cdot DMA\cdot (H_2O)_2$ E= -1048.050313 a.u.

O	-1.49721400	-1.73581300	-0.71292400
S	-0.12511100	-1.18565800	-0.79169400
O	0.31999900	-0.73450300	0.57174700
O	-0.49181600	0.30840800	-1.51044500
H	-1.72955300	0.74548500	-0.42483900
H	2.36787300	1.24091700	-0.74885800
N	-2.52615800	0.79578400	0.25445800
C	-2.54355200	2.10324400	0.93491500
H	-2.32971200	0.03750200	0.95308000
H	-1.58392900	2.25144300	1.42373800
H	-3.34101300	2.11329800	1.67469100
H	-2.70885200	2.88919100	0.20134400
C	-3.76962900	0.46387400	-0.47131100
H	-4.59670900	0.42354000	0.23452300
H	-3.61832900	-0.50214100	-0.94634900
H	-3.95786300	1.22870300	-1.22170900
O	-1.91980200	-1.20299200	2.07748000
H	-2.17701100	-1.93164400	1.49601600
H	-0.98674200	-1.07667700	1.80143500
N	3.08573000	0.26186900	0.44366900
C	3.69008700	-0.91054300	-0.18258500
H	2.20737800	-0.03251400	0.86893000
H	2.96151000	-1.37945000	-0.84352000
H	4.02516300	-1.65190400	0.55196300
H	4.55427900	-0.60458100	-0.77535000
C	3.96443900	0.87593200	1.43102000
H	4.28743700	0.16887800	2.20459500
H	3.45539100	1.71095200	1.90998200
H	4.85615500	1.26116200	0.93369100
O	1.80118200	1.72202500	-1.42891300
H	2.31594700	1.76983800	-2.23787000
H	0.33648200	0.85292700	-1.52307600

36. $NH_2(CH_3)_2^+\cdot HSO_3^-\cdot DMA\cdot (H_2O)_3$ E= -1124.479376 a.u.

O	1.32337600	1.91097000	-0.30072400
S	-0.04885700	1.44150900	-0.66711900
O	-0.57334300	0.93401500	0.81152000
O	0.08841200	0.13124100	-1.40765200
H	1.63854000	-0.60042000	-0.29730100
H	-2.71919400	-1.19672900	-0.03962000
N	1.83669900	-1.01488400	0.62854100
C	0.84071000	-2.07479500	0.89487700
H	1.72287400	-0.23011700	1.30830800
H	-0.14817000	-1.62864100	0.85269300
H	1.03241300	-2.50087400	1.87729700
H	0.92065600	-2.83968700	0.12656500
C	3.24027000	-1.47609200	0.67502700
H	3.44293900	-1.87856100	1.66495700

H	3.88674400	-0.63013600	0.46496000
H	3.38809900	-2.23744400	-0.08615700
O	1.87105300	1.23001200	2.29782200
H	1.80505200	1.71456000	1.44837000
H	0.97100800	1.30528500	2.63517500
N	-2.91835700	-0.27330600	0.33804900
C	-3.45889200	0.55341800	-0.74100800
H	-1.46864800	0.46414900	0.68329600
H	-2.78443900	0.51293800	-1.59588200
H	-3.52218200	1.58924800	-0.40330300
H	-4.45631900	0.22799700	-1.05576100
C	-3.82848500	-0.35915200	1.47593200
H	-3.93939700	0.62978600	1.92254300
H	-3.41647500	-1.02947400	2.22881800
H	-4.82337100	-0.71831800	1.18973400
O	-1.42725800	-2.13612500	-1.41327000
H	-1.60653000	-2.41685900	-2.31331900
H	-0.90909600	-1.30655000	-1.48884400
O	2.93782000	0.16828200	-1.88790800
H	2.15018400	-0.02702800	-2.40944600
H	2.66653300	0.98262900	-1.43257700

37. $NH_2(CH_3)_2^+ \cdot HSO_3^- \cdot DMA \cdot (H_2O)_4$ G= -1200.898107 a.u.

O	1.92499000	-0.05574000	-1.63358900
S	0.49571200	-0.49378300	-1.64184100
O	-0.31582000	0.50675700	-0.85247100
O	0.56145400	-1.74577100	-0.56937100
H	2.35093000	0.29802200	-0.05260300
H	-2.08192600	-1.03692600	1.05993700
N	2.48235400	0.76083700	0.89146600
C	1.67753800	0.04627100	1.90991000
H	2.07953500	1.70001900	0.74884000
H	0.63565100	0.04446700	1.59926800
H	1.79268200	0.55236800	2.86644400
H	2.04419800	-0.97480700	1.97412700
C	3.91991100	0.83200100	1.22328100
H	4.04608300	1.37441000	2.15798700
H	4.44147500	1.34783400	0.42074200
H	4.29241200	-0.18471700	1.31304700
O	0.73891300	2.80165000	0.02936300
H	0.96705800	3.37957800	-0.70408900
H	0.36424500	1.98520000	-0.38427900
N	-3.40477900	-0.20366600	0.52037100
C	-3.60246300	-0.63820100	-0.86267500
H	-3.16331400	0.78582900	0.50222900
H	-2.69905600	-0.43025200	-1.43445200
H	-4.45206200	-0.13743400	-1.33874000
H	-3.78153800	-1.71489300	-0.87999700
C	-4.60259100	-0.40334300	1.33090600
H	-5.48457900	0.08437800	0.90103100
H	-4.43919100	-0.01231200	2.33403300
H	-4.81295800	-1.47166100	1.40946000
O	-1.26523100	-1.58133200	1.33124000
H	-1.58884300	-2.43052700	1.64246200
H	-0.17911200	-1.67878800	0.09843300
O	3.36508200	-2.00917100	-0.00958000

H	2.40975000	-2.15666400	-0.09795800
H	3.58256000	-1.64200800	-0.87363500
O	-2.34170700	2.40733500	-0.49766200
H	-1.75077200	1.72492900	-0.85643400
H	-1.74345700	2.98410100	-0.01122000

38. TS11 G= -1200.894482 a.u.

O	-1.74247700	-1.11087900	1.09607600
S	-0.58470500	-1.77678500	0.37857100
O	0.66760900	-0.97617700	0.73643600
O	-0.82807900	-1.35753100	-1.12780400
H	-1.98796200	0.30692000	0.52026200
H	1.90144000	0.19541100	-1.33196100
N	-2.12938700	1.36808100	0.33270700
C	-1.94205600	1.70331400	-1.09558900
H	-1.36776800	1.80058900	0.87163300
H	-0.96163000	1.35786300	-1.41542800
H	-2.02305400	2.78258400	-1.21817700
H	-2.71800400	1.19714200	-1.66298500
C	-3.45620600	1.74634600	0.85332800
H	-3.59267600	2.82327500	0.77084100
H	-3.52521700	1.43822900	1.89392700
H	-4.20565600	1.22374800	0.26339300
O	0.52404800	1.64771700	1.18485600
H	0.99948200	1.69396400	2.02209900
H	0.46601300	0.67020400	1.01470700
N	2.80752100	0.39383300	-0.58512200
C	3.63933800	-0.81677200	-0.52293400
H	2.38585700	0.53991900	0.33148000
H	3.00288500	-1.64424800	-0.21900400
H	4.44610400	-0.68658400	0.19731500
H	4.05496200	-1.00987400	-1.51142000
C	3.54473400	1.60111700	-0.97758100
H	4.36952900	1.78734900	-0.28951200
H	2.86030300	2.44605400	-0.96736800
H	3.93867900	1.46696600	-1.98395400
O	0.88252900	0.00401600	-2.13386800
H	1.10750100	-0.35882300	-2.99394900
H	0.03692400	-0.71580800	-1.61166100
O	-3.70249900	-0.96627300	-0.99933000
H	-2.82352400	-1.14604800	-1.36989600
H	-3.58851000	-1.30478800	-0.10239200
O	2.50517200	-0.10642500	2.45273200
H	1.81522500	-0.68337900	2.06527700
H	2.85216300	-0.56511500	3.22041800

39. $(NH_2(CH_3)_2^+)_2 \cdot SO_3^{2-} \cdot (H_2O)_4$ E= -1200.893985 a.u.

O	-1.74247700	-1.11087900	1.09607600
S	-0.58470500	-1.77678500	0.37857100
O	0.66760900	-0.97617700	0.73643600
O	-0.82807900	-1.35753100	-1.12780400
H	-1.98796200	0.30692000	0.52026200
H	1.90144000	0.19541100	-1.33196100
N	-2.12938700	1.36808100	0.33270700
C	-1.94205600	1.70331400	-1.09558900
H	-1.36776800	1.80058900	0.87163300

H	-0.96163000	1.35786300	-1.41542800
H	-2.02305400	2.78258400	-1.21817700
H	-2.71800400	1.19714200	-1.66298500
C	-3.45620600	1.74634600	0.85332800
H	-3.59267600	2.82327500	0.77084100
H	-3.52521700	1.43822900	1.89392700
H	-4.20565600	1.22374800	0.26339300
O	0.52404800	1.64771700	1.18485600
H	0.99948200	1.69396400	2.02209900
H	0.46601300	0.67020400	1.01470700
N	2.80752100	0.39383300	-0.58512200
C	3.63933800	-0.81677200	-0.52293400
H	2.38585700	0.53991900	0.33148000
H	3.00288500	-1.64424800	-0.21900400
H	4.44610400	-0.68658400	0.19731500
H	4.05496200	-1.00987400	-1.51142000
C	3.54473400	1.60111700	-0.97758100
H	4.36952900	1.78734900	-0.28951200
H	2.86030300	2.44605400	-0.96736800
H	3.93867900	1.46696600	-1.98395400
O	0.88252900	0.00401600	-2.13386800
H	1.10750100	-0.35882300	-2.99394900
H	0.03692400	-0.71580800	-1.61166100
O	-3.70249900	-0.96627300	-0.99933000
H	-2.82352400	-1.14604800	-1.36989600
H	-3.58851000	-1.30478800	-0.10239200
O	2.50517200	-0.10642500	2.45273200
H	1.81522500	-0.68337900	2.06527700
H	2.85216300	-0.56511500	3.22041800

40. $H_2SO_3{\cdot}O_3$ G= -850.453349 a.u.

O	-2.63124300	-0.19877600	0.05066900
S	-1.29027300	0.12093700	-0.37075800
O	-0.37273700	-1.19286400	-0.08837700
O	-0.74120700	1.05228500	0.84197000
H	0.17918000	1.29823000	0.65568000
H	-0.67840200	-1.61576000	0.73189900
O	2.39165900	-0.21561000	-0.29032200
O	2.22238400	-0.33885600	0.92216100
O	1.77409300	0.69163800	-0.86803200

41. TS12 G= -850.424103 a.u.

O	-2.27834000	-0.05449300	-0.47122900
S	-0.88156400	-0.09761700	-0.17199900
O	-0.63969500	-0.58265200	1.33342000
O	-0.28643600	1.33021800	-0.13338400
H	0.79140500	1.23928300	-0.03284200
H	-0.61331200	0.17669800	1.93953300
O	1.83133700	-0.56640300	0.06531500
O	2.06288800	0.74545800	0.19161900
O	1.05111100	-0.85389100	-0.88007800

42. $HSO_3{\cdot}O_2H$ G= -850.532681 a.u.

O	-1.75093900	0.69156600	-0.78924600
S	-0.73337300	0.15874700	0.02487500
O	-1.18243300	-1.27281800	0.48615400

O	-0.17420700	0.83542000	1.15009600
H	1.93967800	0.79059400	0.83167400
H	-0.69209000	-1.53734500	1.27885300
O	1.56246200	-0.68687000	-0.23156600
O	2.38931500	0.38251000	0.06971200
O	0.46660000	-0.17395900	-0.99871600

43. TS13 G= -850.520756 a.u.

O	1.84877000	-0.53976300	-0.67793700
S	0.70781100	-0.10749100	0.01905200
O	1.05777300	1.23969200	0.73117700
O	-0.00092400	-0.93953300	0.95988800
H	-1.43432500	-0.90248500	0.61860400
H	0.36099400	1.48486300	1.36020600
O	-1.57129000	0.66188900	-0.32533400
O	-2.17977900	-0.53125900	0.03635600
O	-0.37323300	0.31743500	-1.07740700

44. $H_2SO_4 \cdot {}^3O_2$ G= -850.603475 a.u.

O	-2.02783000	-0.35598300	-0.98095200
S	-0.96030700	-0.12366200	-0.08882000
O	-1.60441000	0.33344400	1.28099200
O	-0.24318400	1.19555400	-0.57154700
H	0.69740700	1.15586500	-0.33942300
H	-1.04636100	0.03610600	2.01279000
O	3.10617100	-0.53200300	-0.12169700
O	2.68632100	0.54880800	0.15545000
O	0.04716500	-1.09149200	0.20622400

45. $NH_2(CH_3)_2^+ \cdot HSO_3^- \cdot O_3 \cdot H_2O$ G= -1061.960437 a.u.

C	-3.30340500	-1.34381300	1.04557300
N	-2.54865900	-0.47965200	0.12322700
C	-3.19384200	-0.29185300	-1.19272000
H	-4.28632100	-0.91441400	1.23043900
H	-2.75515400	-1.42764400	1.98036400
H	-3.41325500	-2.32990800	0.59954600
H	-2.38567300	0.46013800	0.54165600
H	-2.53622100	0.32995300	-1.79541000
H	-4.16177400	0.18765700	-1.06057000
H	-3.32049200	-1.26450000	-1.66366800
O	0.64935800	0.62397000	1.04686900
H	1.40211600	0.19300500	1.47407000
H	-1.55665600	-0.87120600	-0.05517000
O	-0.26753200	1.11402600	-1.12604900
S	0.63631700	0.08912500	-0.54998300
O	-0.09634100	-1.21307200	-0.42835500
O	3.83835700	-0.51382300	-0.11022900
O	3.66617600	0.70769000	-0.08865400
O	3.10904300	-1.20625000	0.61907600
O	-1.87523700	2.13972300	0.82906200
H	-1.26195600	2.13159800	1.57236700
H	-1.27218000	2.03275000	0.06264500

46. TS14 G= -1061.948288 a.u.

C	2.80741400	-1.88007600	-0.56178900
N	2.20733500	-0.68162000	0.05679600

C	2.99623700	-0.11500100	1.17545500
H	3.77929700	-1.62008000	-0.97514200
H	2.15011700	-2.23545100	-1.35084800
H	2.92287000	-2.65149900	0.19566300
H	2.08650200	0.07616700	-0.65365200
H	2.44150500	0.73009800	1.57654900
H	3.96421200	0.20807700	0.79926800
H	3.12392400	-0.87696000	1.94061800
O	-0.73039700	0.44209900	-1.09898800
H	-1.49829600	-0.18238000	-1.30604300
H	1.24118300	-0.88142200	0.41736800
O	0.23458400	1.69785100	0.72043000
S	-0.74745800	0.62751600	0.50479100
O	-0.31367700	-0.67625700	1.04443300
O	-3.05688400	-0.88794000	0.19470500
O	-2.95472000	0.33770400	0.48196300
O	-2.75761000	-1.17443900	-1.06337700
O	1.92988000	1.60024300	-1.50833700
H	1.26060700	1.43500400	-2.18328300
H	1.40475400	1.98589500	-0.78334600

47. $NH_2(CH_3)_2^+ \cdot [SO_3 \cdot O_3H]^- \cdot H_2O$ G= -1062.055615 a.u.

C	3.45816000	-0.52472500	-1.33015000
N	2.50772700	-0.31857700	-0.21849100
C	2.99797600	-0.80249500	1.09271700
H	4.38189500	0.00742200	-1.11526300
H	3.01568200	-0.14563000	-2.24734500
H	3.65966100	-1.58840200	-1.43174600
H	2.25603200	0.69193700	-0.14170700
H	2.21039200	-0.64049400	1.82362100
H	3.89623600	-0.25157100	1.36225500
H	3.22302200	-1.86341400	1.01265200
O	-1.11869200	1.10888000	-0.60627400
H	-3.12613200	0.87880200	-0.86236800
H	1.58600100	-0.77785400	-0.42873900
O	-0.00613900	0.28048100	1.41055200
S	-0.71209800	-0.03715700	0.18635000
O	-0.05225300	-1.07454600	-0.60064000
O	-2.95942700	-0.89573900	-0.35264200
O	-2.09282600	-0.69494800	0.76024400
O	-3.75183600	0.24445600	-0.46678200
O	1.50513000	2.28922000	0.06658200
H	0.77649100	2.36165800	-0.56685700
H	1.03173100	2.05300000	0.87961300

48. TS15 G= -1062.042772 a.u.

C	3.37198800	-0.89224900	-1.29489700
N	2.49289100	-0.36689700	-0.23170000
C	2.97671500	-0.64427900	1.14011600
H	4.35439400	-0.43269600	-1.21211600
H	2.93258800	-0.66344700	-2.26207700
H	3.45909100	-1.97007800	-1.17978100
H	2.35818300	0.65819700	-0.33710100
H	2.25179600	-0.23583800	1.83868400
H	3.95021200	-0.17878500	1.27755300
H	3.05623000	-1.72095800	1.27157600

O	-1.33129900	0.92836800	-0.73821500
H	-2.47780500	0.76286200	-0.89535300
H	1.51705600	-0.76364600	-0.33480200
O	0.05423200	0.64458200	1.22841100
S	-0.78838500	-0.06306800	0.28010500
O	-0.06106800	-1.12021800	-0.41877300
O	-3.17270800	-0.87418700	-0.28611900
O	-1.99547200	-0.62278600	1.01252600
O	-3.58991300	0.27574600	-0.63348200
O	1.75814700	2.32554900	-0.12733100
H	1.36096500	2.85840800	-0.82181800
H	1.01362500	2.02610200	0.42801700

49. $NH_2(CH_3)_2^+ \cdot HSO_4^- \cdot {}^3O_2 \cdot H_2O$ G= -1062.121916 a.u.

C	3.48109400	-0.79830500	-1.44718000
N	2.65697200	-0.33792000	-0.31460600
C	3.19643800	-0.71187200	1.01109300
H	4.47649500	-0.36478500	-1.37542900
H	3.00811600	-0.49232900	-2.37659900
H	3.55011600	-1.88322800	-1.41509400
H	2.52566300	0.69114000	-0.34565300
H	2.50760500	-0.34305500	1.76598300
H	4.18274700	-0.27053900	1.13905900
H	3.26311800	-1.79613600	1.06751800
O	-1.05211400	0.95246200	-0.67577200
H	-1.98563300	0.80994400	-0.88187000
H	1.66261800	-0.72944800	-0.39676500
O	0.28214400	0.54868400	1.28072300
S	-0.60319700	-0.18436500	0.38356400
O	0.16153000	-1.15352700	-0.41753600
O	-4.66661100	-0.52359600	-0.11744400
O	-1.81426200	-0.70290600	0.94482200
O	-4.15225400	0.28873300	-0.82155800
O	1.88958400	2.33433200	0.00542000
H	1.42578900	2.87046100	-0.64326500
H	1.18637800	1.93085600	0.55460200

50. $NH_2(CH_3)_2^+ \cdot HSO_3^- \cdot O_3 \cdot (H_2O)_2$ G= -1138.384992 a.u.

C	2.44994900	-0.04954700	1.46162500
N	2.62714700	0.01346700	-0.00729800
C	3.72336300	-0.84132200	-0.50562700
H	2.27783900	-1.08630400	1.73712100
H	1.58662800	0.55692200	1.72358000
H	3.34822500	0.33339300	1.94127800
H	1.71561600	-0.25298400	-0.47448400
H	3.78395700	-0.74266400	-1.58662100
H	3.49393700	-1.86990300	-0.23932300
H	4.66034000	-0.52524100	-0.05154500
O	-0.78075800	-0.74712900	0.88858400

H	-1.58906300	-0.57136100	1.38975000
H	2.75796500	1.00449200	-0.26978600
O	0.26019000	-0.37184200	-1.24848300
S	-0.79273500	0.30478500	-0.43144200
O	-0.25111800	1.55348200	0.15578200
O	-4.07187200	0.16115500	-0.14910800
O	-3.66019300	-0.93524300	-0.53562000
O	-3.52663100	0.65781500	0.85048200
O	1.19056400	-2.73247900	0.03860200
H	0.52398300	-2.44764900	0.67668300
H	0.84858400	-2.32651600	-0.76975400
O	2.15041600	2.71758000	-0.23380600
H	1.21495200	2.43115700	-0.13029600
H	2.14611700	3.44434500	-0.85990600

51. TS16 G=-1138.373949 a.u.

C	-0.90196900	2.36641300	0.35978100
N	-1.67955800	1.20155100	-0.12652300
C	-2.92720600	1.56108600	-0.83182500
H	-0.64008300	2.99640700	-0.48775600
H	0.00204800	1.99828100	0.83691400
H	-1.51547700	2.92344200	1.06397100
H	-1.06962300	0.63962100	-0.73787600
H	-3.41643100	0.64307900	-1.14307000
H	-2.68577400	2.17516000	-1.69642700
H	-3.56494600	2.11913100	-0.15042300
O	0.64969300	-0.09151700	-1.28703200
H	1.29377500	0.66093700	-1.18255300
H	-1.90806100	0.56641700	0.68151000
O	0.22293600	-2.30532200	-0.39703600
S	0.98183600	-1.10635100	-0.03681700
O	0.46792100	-0.40010600	1.14505600
O	2.94280400	0.82914300	0.48016000
O	3.11142400	-0.31044600	-0.02281600
O	2.37038500	1.69871600	-0.32920500
O	-2.41769300	-1.66229500	-0.72891200
H	-2.90293900	-2.28704400	-1.27391900
H	-1.49695100	-2.00017500	-0.69640700
O	-2.24997300	-0.59033700	1.87090700
H	-1.30871700	-0.78817200	2.00613600
H	-2.54420700	-1.26401400	1.23792400

52. $NH_2(CH_3)_2^+ \cdot [SO_3 \cdot O_3H]^- \cdot (H_2O)_2$ G= -1138.481984 a.u.

C	2.16321100	-0.38187300	1.73064500
N	2.43055900	-0.24899600	0.27738900
C	3.86336600	-0.37730100	-0.06530900
H	2.51926700	-1.35423100	2.06364600
H	1.09356600	-0.29071600	1.89732900
H	2.69315900	0.41032500	2.25476700
H	1.89385300	-0.97949000	-0.24442900
H	3.97916000	-0.27481800	-1.14100500
H	4.21901600	-1.35432800	0.25274200
H	4.41818200	0.40890600	0.44106000
O	-0.89218800	-1.04113400	0.45756300
H	-2.87228100	-1.40300800	0.78028100
H	2.10225500	0.68888700	-0.04857500

O	-0.14396900	0.30586400	-1.44149800
S	-0.84902700	0.27520300	-0.17853000
O	-0.47452200	1.32661800	0.74565900
O	-3.22615000	0.39126900	0.47898900
O	-2.38531100	0.56780200	-0.65700700
O	-3.66381200	-0.93166200	0.46237900
O	1.09516500	-2.29958400	-1.05888800
H	0.29911500	-2.23170800	-0.50264100
H	0.81122100	-1.84891500	-1.86471100
O	1.79167500	2.36283100	-0.44427000
H	0.95206600	2.33754100	0.05223500
H	1.49537200	2.33029200	-1.36137800

53. TS17 G=-1138.470802 a.u.

C	1.79424400	0.11316600	2.18984700
N	2.02173300	-0.33205100	0.79477000
C	3.24830400	-1.13271000	0.60205500
H	1.73992200	-0.75964700	2.83633300
H	0.85794000	0.66418200	2.21536000
H	2.62193000	0.75059300	2.49182700
H	1.18760900	-0.85766800	0.48921000
H	3.30986500	-1.40938700	-0.44647300
H	3.20141100	-2.02138800	1.22723300
H	4.10787500	-0.52880300	0.88305400
O	-0.64365100	-1.08784500	0.22671200
H	-1.70908100	-1.54708800	0.96879200
H	2.06357700	0.51222300	0.16543800
O	-0.97014200	0.18442100	-1.84292400
S	-1.05956800	0.19579900	-0.40608300
O	-0.43388300	1.32168300	0.24646400
O	-2.80985300	-0.10742500	1.27383200
O	-2.64780100	0.22656900	-0.12431900
O	-2.66963200	-1.49398200	1.36747200
O	1.65454600	-0.60281100	-2.30852100
H	1.87413200	-1.06737300	-3.12021500
H	0.68799900	-0.47016700	-2.32818200
O	2.11702100	1.80040900	-0.92258100
H	1.20823800	2.09999500	-0.76637400
H	2.06544600	1.25737800	-1.72528700

54. $NH_2(CH_3)_2^+ \cdot HSO_4^- \cdot {}^3O_2 \cdot (H_2O)_2$ G= -1138.522339 a.u.

C	1.66227300	-0.33210000	1.93136500
N	2.12494300	-0.32381200	0.52466900
C	3.57977000	-0.54549300	0.37245700
H	1.87255600	-1.30552700	2.36842600
H	0.59643600	-0.12441900	1.93790100
H	2.19951800	0.44412600	2.47195000
H	1.63118400	-1.03405100	-0.03661800
H	3.80932100	-0.59537400	-0.68772400
H	3.84804600	-1.48495800	0.84994200
H	4.10786600	0.28037400	0.84297900
O	-0.70125000	-1.25984200	-0.04979000
H	-1.54105400	-1.54362000	0.36149500
H	1.89403400	0.60969800	0.10764600
O	0.08371000	0.25485300	-1.72889700

S	-0.96798400	0.21416600	-0.71817900
O	-0.70829900	1.15255200	0.37029600
O	-3.58426300	0.65594300	1.67789500
O	-2.31985700	0.18126100	-1.18915000
O	-3.03578200	-0.38646200	1.85949000
O	1.75622100	-1.90789700	-1.78033900
H	1.33911000	-2.75692600	-1.94589300
H	1.08445400	-1.24004400	-2.02009400
O	1.69488700	2.28653400	-0.28320700
H	0.76192900	2.19349900	-0.00398800
H	1.63041500	2.27559200	-1.24518900

55. $NH_2(CH_3)_2^+\cdot HSO_3^-\cdot NO_2\cdot H_2O$ G= -1041.626250 a.u.

O	-1.14066400	-1.83791300	0.10632100
S	0.27831400	-1.60343400	-0.21497000
O	0.88631400	-0.65021600	0.76171300
O	0.03374100	-0.52077400	-1.56352000
H	-1.26408300	0.39616300	-0.80667600
H	0.88574700	-0.09732100	-1.74019600
N	-2.00499400	0.82269400	-0.21083700
C	-1.93945100	2.29450900	-0.28049500
H	-1.76076300	0.51717100	0.76508300
H	-0.94266700	2.61145000	0.01608300
H	-2.67373500	2.71696700	0.40150800
H	-2.14754300	2.61961100	-1.29749600
C	-3.31287400	0.25017300	-0.59310600
H	-4.07601200	0.62544300	0.08542600
H	-3.22776100	-0.83055300	-0.52149700
H	-3.54739000	0.54646900	-1.61332100
O	-1.13820200	0.23145600	2.32720800
H	-1.54110600	-0.59480800	2.61445800
H	-0.29073000	-0.06579000	1.91807900
N	2.89282000	0.95445700	-0.01576600
O	3.68399700	0.81138700	0.84143700
O	2.85383800	0.65380900	-1.16742100

56. TS18 G= -1041.605625 a.u.

O	0.10419600	-1.79596400	0.27042800
S	1.36481200	-1.39608500	-0.38805500
O	2.29532800	-0.65713800	0.46584200
O	0.86459200	-0.26593600	-1.47506500
H	-0.89996000	0.04048400	-0.77119100
H	1.30831200	0.60168100	-1.22474500
N	-1.83682500	0.14337200	-0.34979200
C	-2.37745200	1.49026100	-0.62484900
H	-1.66687700	0.01596800	0.67707700
H	-1.68301100	2.22909700	-0.23496400
H	-3.34265600	1.59078100	-0.13408200
H	-2.49469500	1.61610000	-1.69868400
C	-2.70068900	-0.96187500	-0.82310100
H	-3.65986000	-0.89897600	-0.31469700
H	-2.20235700	-1.89747200	-0.58657100
H	-2.84040200	-0.86782800	-1.89766400
O	-1.30978300	-0.52835700	2.28335500
H	-0.64438300	-1.12436200	1.89251700

H	-0.78522300	0.17580900	2.68092500
N	1.13264000	2.20192800	0.49311000
O	0.44846500	1.33118900	0.99401200
O	1.65623600	2.07478900	-0.60039300

57. $NH_2(CH_3)_2^+ \cdot SO_3^- \cdot HNO_2 \cdot H_2O$ G= -1041.636501 a.u.

O	-2.39206300	-0.69968100	-0.59368200
S	-1.27221200	-1.43878000	0.01375100
O	-0.86486800	-0.92009200	1.33063100
O	-0.11564500	-1.54595100	-0.91729800
H	0.45564700	0.58465100	-0.03912300
H	1.42855800	-1.78335400	-0.34770500
N	0.03205800	1.49725700	-0.25270900
C	0.93919400	2.54340200	0.27119400
H	-0.88566400	1.53564100	0.25598800
H	1.06283300	2.39286800	1.34041800
H	0.50121900	3.51913500	0.07697100
H	1.90429600	2.45065200	-0.22116000
C	-0.19686900	1.57929100	-1.71584300
H	-0.61731100	2.55525300	-1.94674600
H	-0.88304400	0.78535900	-1.99891000
H	0.75691800	1.44923700	-2.22230900
O	-2.38690400	1.53447700	1.09157200
H	-2.79882500	0.88425300	0.49572500
H	-2.13516300	0.98116600	1.84348500
N	2.38144900	-0.31108100	0.19269500
O	3.42475400	0.08586500	0.57555500
O	2.36190400	-1.62633900	-0.03286200

58. $NH_2(CH_3)_2^+ \cdot HSO_3^- \cdot NO_2 \cdot (H_2O)_2$ G= -1118.052845 a.u.

O	-0.99182900	-1.44270300	0.98815300
S	0.43583700	-1.72859600	0.68382500
O	1.31098600	-0.66786400	1.24252200
O	0.37280700	-1.26970300	-0.97709100
H	-1.29404700	0.07579900	-0.02371900
H	1.27203800	-1.30922700	-1.32951700
N	-1.52231900	1.08644600	-0.05855600
C	-1.36848900	1.60433300	-1.43185400
H	-0.85000600	1.51741800	0.60800600
H	-0.36379700	1.37424300	-1.77410500
H	-1.52975600	2.68008700	-1.42705200
H	-2.10189000	1.10903400	-2.06264800
C	-2.89009100	1.22965700	0.48427400
H	-3.15282600	2.28438800	0.52450500
H	-2.90350000	0.79585100	1.48105300
H	-3.57022700	0.68685800	-0.16744900
O	0.24084600	1.69182300	1.98941900
H	-0.03573200	1.64649500	2.90795800
H	0.69626400	0.83473500	1.80744800
N	2.80237800	0.90539500	-0.53419100
O	1.88740800	1.64763500	-0.61356400
O	3.14324900	-0.05485700	-1.13706800
O	-2.52642000	-1.37113800	-1.40683600
H	-2.38802700	-1.75166600	-0.52730100

H	-1.65719000	-1.49885800	-1.80794100

59. TS19 G=-1118.037716 a.u.

O	-0.15816000	-1.47487100	0.55266100
S	-1.55655700	-1.39309100	0.06989300
O	-1.64779200	-0.80492300	-1.28537600
O	-2.28082900	-0.33242400	1.01735400
H	1.47776300	0.37793400	0.75017800
H	-2.30845300	0.57317100	0.55382600
N	2.16413600	0.06617800	0.04284400
C	2.99240100	1.20850200	-0.39367000
H	1.61494300	-0.27692000	-0.77396500
H	2.33310300	1.96157100	-0.81533500
H	3.69601300	0.86497900	-1.14838900
H	3.52976000	1.60758200	0.46375400
C	2.93538600	-1.05089900	0.62744200
H	3.65020500	-1.41269800	-0.10851000
H	2.23474400	-1.83623300	0.89647800
H	3.45621800	-0.69313700	1.51246200
O	0.90743600	-0.56810700	-2.34606100
H	0.99769100	-1.40064900	-2.81703200
H	-0.02874400	-0.54447900	-2.05748000
N	-1.06960600	2.24930700	-0.44747800
O	-0.19435100	1.41127600	-0.44101300
O	-2.19789800	2.02479400	-0.03910100
O	0.67678600	0.38615100	2.43119600
H	0.11033200	-0.30826200	2.05065300
H	0.07136000	1.08741700	2.68760700

60. $NH_2(CH_3)_2^+ \cdot SO_3^- \cdot HNO_2 \cdot (H_2O)_2$ G= -1118.060686 a.u.

O	-0.04988800	1.81202700	-0.25535000
S	-1.43621600	1.41072900	0.06300700
O	-1.58599400	0.81164500	1.39894300
O	-2.04192600	0.58771100	-1.01036300
H	1.39438100	-0.22067400	-0.79208700
H	-2.25721500	-0.98802500	-0.47577200
N	2.09966500	-0.16313200	-0.03363100
C	2.84186100	-1.43870400	0.05540000
H	1.58615100	0.00039900	0.86029300
H	2.13461600	-2.23109700	0.27966900
H	3.58233300	-1.36024800	0.84806800
H	3.33414600	-1.62666900	-0.89623300
C	2.96177300	1.00429500	-0.31938100
H	3.71922900	1.08525100	0.45728700
H	2.33471200	1.89098600	-0.33332300
H	3.43434400	0.86113400	-1.28846900
O	0.86375100	0.13981000	2.42268100
H	1.09865600	0.83305100	3.04498300
H	-0.06098100	0.33588200	2.16008100
N	-1.19377800	-2.43047000	0.18157400
O	-0.28564600	-1.66182100	0.07825100
O	-2.36673200	-1.94625900	-0.19079300
O	0.63996000	0.14071500	-2.41229300
H	0.26751300	0.94627100	-2.01710300
H	-0.13964600	-0.40688100	-2.55582300

61. $NH_2(CH_3)_2^+ \cdot HNO_2 \cdot NO_2 \cdot H_2O$ G= -1246.83518908 a.u.

C	-0.42465500	1.59791600	1.73783200
N	-0.05091000	1.49143300	0.30921400
C	0.57302700	2.72080400	-0.23854100
H	0.46070000	1.86998900	2.30797400
H	-0.81456000	0.64230700	2.07665200
H	-1.18017400	2.37327500	1.84137100
H	0.59207000	0.69363600	0.14217200
H	0.76570900	2.56002100	-1.29648700
H	1.49728400	2.91185400	0.30122600
H	-0.11999800	3.54826100	-0.10701800
O	-0.71385500	-1.18652600	-2.50135200
H	-1.57828100	-1.79731300	-1.23281400
H	-0.87521300	1.26148300	-0.28079400
O	0.43489300	0.92751000	-2.99483300
S	-0.86129400	0.26112600	-2.79280200
O	-1.72987400	0.94416600	-1.81224500
N	-1.42018000	-1.24687900	0.52506700
O	-1.93980600	-2.10550600	-0.35599300
O	-1.76253700	-1.46782900	1.63519700
O	1.66718300	-0.44993700	-0.85306900
H	1.64766400	-0.00832300	-1.71886600
H	1.14731600	-1.24863400	-1.01180700
N	2.58350900	-0.28060100	1.82344800
O	1.60788600	-0.92850100	1.97271200
O	2.78390400	0.85938400	1.56974300

62. $NH_2(CH_3)_2^+ \cdot HSO_4^- \cdot (HNO_2)_2$ G= -1246.91002977 a.u.

C	-0.80285600	2.35899800	1.18326600
N	0.11923700	1.77910800	0.17932000
C	0.70720800	2.75599300	-0.76237600
H	-0.26393900	3.10126800	1.76723000
H	-1.15061900	1.54709500	1.81554500
H	-1.63833600	2.81626300	0.65924200
H	0.86073900	1.24439300	0.64957800
H	1.33926300	2.21689100	-1.46318500
H	1.29669300	3.47831100	-0.20336500
H	-0.10019200	3.25777600	-1.29006200
O	-1.21198100	-2.30604100	-0.00892600
H	-2.49913600	-1.38281600	-0.59650200
H	-0.36882000	1.04372900	-0.35404100
O	-0.00480600	-0.69108900	1.38183200
S	-0.03737100	-1.47004400	0.16145500
O	0.28882600	-0.69213900	-1.02907700
N	-2.55479200	0.46482800	-0.62546000
O	-3.15309900	-0.69288700	-0.89935900
O	-3.19942800	1.40657700	-0.94062400
O	1.21703300	-2.46371900	0.37049000
H	2.49730600	-0.72153900	0.76450000
H	1.39464900	-2.92366700	-0.46185900
N	3.26862900	0.23811400	-0.70700600
O	3.44246300	-0.82346300	-1.16483200
O	2.72017500	0.21358300	0.55756700

63. $NH_2(CH_3)_2^+ \cdot HSO_3^- \cdot N_2O_5$ G= -1245.447442 a.u.

S	0.38053900	-1.83189100	-0.37142200
O	0.55362300	-1.43501900	1.27227100
O	-0.21847800	-0.57786500	-0.89996000
O	1.84239300	-1.86288700	-0.70297200
H	-0.33319100	-1.28273600	1.62764300
H	2.31938100	-0.51033800	-0.33414900
N	2.45307200	0.51335300	0.05060400
H	1.65160000	0.60627800	0.68126400
C	3.71200900	0.61963100	0.80860000
H	4.54284200	0.44260800	0.12948100
H	3.80196400	1.61155800	1.24743800
H	3.71039600	-0.13795400	1.58842600
C	2.31234400	1.47921000	-1.05691000
H	2.33505200	2.49466800	-0.66668200
H	3.13275800	1.32758600	-1.75497100
H	1.36282000	1.28046300	-1.54943600
O	-1.38910700	2.05355400	-0.80485600
O	-2.68831200	-1.28919100	0.64190000
N	-2.65494300	-0.24591900	0.08844200
N	-1.04147400	1.64426500	0.24247900
O	-1.86561200	0.70415000	0.93906900
O	-3.14990800	0.17277500	-0.88455000
O	-0.09146700	1.92238100	0.91321500

64. TS20 G= -1245.419152 a.u.

S	0.26270100	-1.47430200	0.12122600
O	-0.34089000	-1.05050000	-1.36650900
O	-0.35349200	-0.45760200	1.03080200
O	-0.27473400	-2.80934100	0.29509100
H	0.14190400	-0.24993500	-1.64045400
H	-2.21917900	0.56095200	-0.99378100
N	-2.56745800	0.31911400	-0.06486800
H	-1.66160400	0.07776000	0.47107400
C	-3.24367900	1.47865000	0.55022100
H	-4.14659600	1.71661900	-0.00845300
H	-3.50217600	1.22060000	1.57438700
H	-2.55531100	2.31924900	0.53904700
C	-3.38200400	-0.91490900	-0.12384200
H	-3.64913300	-1.19294400	0.89286500
H	-4.27988900	-0.73533900	-0.71127300
H	-2.77595400	-1.70191500	-0.56729400
O	1.11567700	2.42221100	0.66582600
O	3.22343200	-0.68040500	-0.56717400
N	2.53295600	-0.19656900	0.21779100
N	0.71436100	1.84548900	-0.31169000
O	1.51131400	0.98726100	-0.91462600
O	2.44153800	0.14503100	1.30703000
O	-0.39287000	1.99523400	-0.81827100

65. $NH_2(CH_3)_2^+ \cdot [SO_3 \cdot NO_2]^- \cdot HNO_3$ G= -1245.517997 a.u.

S	0.95773400	0.49726700	-0.04708100
O	0.56337200	1.88351400	-0.12298600
O	0.64399700	-0.18242400	1.19048500
O	0.75588600	-0.28735900	-1.24439100

H	-1.04757100	2.15936300	-0.01715100
H	-0.58936900	-1.64502900	-0.83303400
N	-0.71917700	-2.25134800	-0.00970000
H	-0.67459000	-1.56678200	0.75922000
C	-2.01946400	-2.95729000	-0.04351500
H	-2.03061600	-3.62610700	-0.90031200
H	-2.12859900	-3.53107700	0.87330400
H	-2.81008700	-2.21749700	-0.12617400
C	0.46953300	-3.13799000	0.09571000
H	0.39340400	-3.71135300	1.01598500
H	0.48118200	-3.80391900	-0.76318800
H	1.35758500	-2.51074800	0.10727400
O	-3.89418700	1.34446700	0.10707200
O	3.34402600	1.65135000	0.03544400
N	2.81590500	0.57567400	0.03645500
N	-2.71018300	1.24724100	0.01617200
O	-2.02175300	2.39427400	0.06091000
O	3.34473100	-0.50963100	0.08985500
O	-2.07123800	0.21425800	-0.11342500

66. $NH_2(CH_3)_2^+ \cdot HSO_3^- \cdot N_2O_5 \cdot H_2O$ G= -1321.870201 a.u.

S	-0.35879800	-1.58837700	0.78600200
O	-0.58077700	-1.47118900	-0.90864000
O	0.45998100	-0.37606200	1.03408200
O	-1.77757100	-1.33009400	1.17996200
H	0.28669300	-1.57549600	-1.32415000
H	-2.14986900	0.14350400	0.60359900
N	-2.06603800	1.10975500	0.16190000
H	-1.36189200	0.95491300	-0.56639900
C	-3.33943900	1.56677200	-0.43929000
H	-4.06478400	1.69836500	0.36027200
H	-3.17302400	2.51789300	-0.94067700
H	-3.68691200	0.80393700	-1.12963500
C	-1.51433800	2.04634700	1.16974800
H	-1.29835300	2.99892000	0.69158500
H	-2.25884500	2.17948900	1.95113900
H	-0.60993100	1.60450600	1.57982000
O	1.99569600	1.99529200	0.42189500
O	2.65014000	-1.68922800	-0.54331600
N	2.82177100	-0.58117400	-0.16919900
N	1.49910700	1.49806100	-0.52281100
O	2.10142000	0.33976200	-1.10671900
O	3.46171900	-0.10371200	0.68463000
O	0.54792700	1.82682500	-1.16827000
O	-3.44766200	-1.48933800	-1.17874500
H	-3.33180300	-1.76935300	-0.26171000
H	-2.55869900	-1.62589000	-1.53088100

67. TS21 G= -1321.849780 a.u.

S	-0.32357300	1.39626200	0.08143900
O	0.28119000	0.87585100	-1.37352500
O	0.14125800	0.37971900	1.04587700
O	0.36054600	2.68568100	0.21780300
H	-0.24995800	0.10054200	-1.62595000
H	2.72345000	0.27735900	-0.05260800
N	2.43193200	-0.72110700	0.03556000

H	1.40321100	-0.67870300	0.08763800
C	2.84447300	-1.50264900	-1.14562900
H	3.93018500	-1.50578400	-1.21140200
H	2.46510500	-2.51581300	-1.04315100
H	2.41249100	-1.03891800	-2.02863700
C	2.91146800	-1.25652400	1.32743200
H	2.51756400	-2.26237000	1.45342400
H	3.99871200	-1.26921200	1.32962600
H	2.53529100	-0.60881000	2.11460500
O	-1.69515900	-2.28796600	0.82782900
O	-3.30801000	0.95265400	-0.75460100
N	-2.73061800	0.44579300	0.10515900
N	-1.15975900	-1.83022600	-0.14728500
O	-1.80984800	-0.93936400	-0.86898300
O	-2.75406000	0.17640200	1.21913700
O	-0.03986900	-2.13048100	-0.55017100
O	3.02225800	1.98721100	0.11173100
H	2.11758200	2.35527700	0.22802200
H	3.41055000	2.50239600	-0.60023400

68. $NH_2(CH_3)_2^+ \cdot [SO_3 \cdot NO_2]^- \cdot HNO_3 \cdot H_2O$ G= -1321.945681 a.u.

S	1.20554300	0.29980700	-0.12748200
O	1.11322900	1.68135200	-0.54043800
O	0.60425500	-0.00192500	1.15561000
O	0.98038300	-0.68992100	-1.15341200
H	-0.28172400	2.37203500	-0.08658200
H	-1.44797700	-1.64207000	-0.60305000
N	-1.32399300	-1.88758400	0.40537000
H	-0.78263000	-1.10637600	0.81243700
C	-2.64918300	-1.99373200	1.05313900
H	-3.20913600	-2.79555000	0.57783100
H	-2.51299800	-2.21285700	2.10934700
H	-3.16855500	-1.04701500	0.92770500
C	-0.49371100	-3.11205900	0.49956700
H	-0.34125900	-3.35503500	1.54829600
H	-1.00869700	-3.92567000	-0.00538900
H	0.46126400	-2.90843200	0.02133400
O	-3.14907900	2.17916500	0.63590900
O	3.73314800	1.02817500	0.13129600
N	3.01611900	0.07297900	0.23704600
N	-2.09670900	1.88464700	0.15806600
O	-1.12426800	2.79470200	0.26941500
O	3.31393400	-1.04859100	0.56388700
O	-1.82865800	0.84037100	-0.42023600
O	-1.48114000	-1.34137800	-2.31768200
H	-0.57133300	-1.00197600	-2.30744000
H	-2.03062400	-0.56510300	-2.46916500

69. $NH_2(CH_3)_2^+ \cdot HSO_3^- \cdot N_2O_5 \cdot (H_2O)_2$ G= -1474.720636 a.u.

S	-0.56318200	-1.57427700	1.21127700
O	-0.66268000	-2.46898100	-0.22099900
O	0.11495100	-0.36812700	0.63402900
O	-1.98793800	-1.25755100	1.48602900
H	-0.78836000	-3.39503100	0.02215700
H	-2.38678600	0.33933800	-1.11995800
N	-1.56359200	0.95483700	-1.29494500

H	-1.04874800	0.93666400	-0.40083800
C	-0.75663400	0.31472200	-2.35628800
H	-1.38036100	0.19417500	-3.23994600
H	0.09739600	0.94644400	-2.58270300
H	-0.41677200	-0.65138400	-1.99018000
C	-1.99014800	2.33451000	-1.60999900
H	-1.10212100	2.94468700	-1.75804900
H	-2.58654500	2.31891700	-2.51948200
H	-2.56831800	2.70592000	-0.77017000
O	2.69845600	1.32737400	0.85702100
O	2.15305700	-1.76015700	-1.53520000
N	2.45502100	-1.04676700	-0.64939300
N	2.01194200	1.35461500	-0.10681300
O	2.22050700	0.36759900	-1.11574300
O	2.90660800	-1.21212300	0.41731100
O	1.17465600	2.13377600	-0.44269300
O	-3.38859000	-1.08172400	-0.85644400
H	-3.02610300	-1.83953900	-1.32652700
H	-3.04822100	-1.20459300	0.05651800
O	-2.24478700	1.63905200	1.35536900
H	-1.52064700	1.96383700	1.91152700
H	-2.33361500	0.71019000	1.61764000
O	0.24261400	1.62433100	2.67985400
H	1.10501900	2.03841100	2.76264800
H	0.35736300	0.89844600	2.04446500

70. TS22 G= -1474.701233 a.u.

S	0.37362500	-1.21122500	-0.49320400
O	0.12554700	-0.17843100	-1.75646000
O	-0.07067600	-0.42860800	0.68911600
O	-0.51587700	-2.31669900	-0.83756600
H	0.83416500	0.48864000	-1.70847600
H	-2.27447900	0.78874100	-0.68251500
N	-1.82319800	1.64130900	-0.24196100
H	-0.89042600	1.32790200	0.05332600
C	-1.68940200	2.69535000	-1.26734900
H	-2.68319800	2.98524300	-1.60056900
H	-1.16438600	3.54534100	-0.83942500
H	-1.12075500	2.29255200	-2.10122800
C	-2.59112900	2.05907400	0.95100600
H	-2.09210300	2.90924300	1.41131300
H	-3.59475300	2.33961000	0.63942800
H	-2.63113400	1.22473000	1.64785900
O	2.38428100	1.62297200	1.48886600
O	3.45595000	-1.34904000	-0.86683500
N	2.87289900	-0.96634700	0.05124000
N	1.92345800	1.61829500	0.37786800
O	2.45215200	0.81499700	-0.52563700
O	2.78075200	-1.03571400	1.19365800
O	0.99075300	2.31292100	-0.01030900
O	-2.95711000	-0.47737200	-1.44912700
H	-2.23029600	-0.87096700	-1.94952400
H	-3.16927600	-1.16394900	-0.77197300
O	-3.10339000	-2.40887100	0.44118000
H	-2.92357500	-1.99501900	1.30081900
H	-2.23149700	-2.70119900	0.13402400

O	-2.03680000	-0.86211200	2.56879800
H	-1.74657200	-1.14629900	3.43885600
H	-1.23327700	-0.77665600	2.02191000

71. $NH_2(CH_3)_2^{+}\cdot[SO_3\cdot NO_2]^{-}\cdot HNO_3\cdot(H_2O)_2$ G= -1474.795320 a.u.

S	0.61844200	1.06441200	-0.41153300
O	-0.44855400	0.47961300	-1.17973900
O	0.91848000	0.37077100	0.82704200
O	1.73449100	1.60809200	-1.12390500
H	-2.47542000	1.38287700	-0.17697000
H	0.63013400	-1.93873600	-0.63579500
N	-0.09382700	-2.18813300	0.08949800
H	-0.42547500	-1.29649300	0.46936500
C	-1.22497700	-2.87742700	-0.56778700
H	-0.86657500	-3.81322000	-0.98979400
H	-2.00884500	-3.05831000	0.16360300
H	-1.60396500	-2.23269300	-1.35768900
C	0.54927900	-2.96498000	1.17239400
H	-0.18817500	-3.17133900	1.94474500
H	0.92595300	-3.89643200	0.75645900
H	1.37120200	-2.37869600	1.57857400
O	-3.95119700	-1.16561100	-0.04228100
O	-1.42264300	2.64381900	0.30132800
N	-0.20275300	2.60766900	0.28938700
N	-3.14368000	-0.32609200	0.23030400
O	-3.27360800	0.83744800	-0.41830100
O	0.52266400	3.45109900	0.72308800
O	-2.23041400	-0.42411500	1.02952500
O	1.77377400	-1.64722200	-1.79066300
H	1.45632500	-1.03690600	-2.46491200
H	2.60310300	-1.23882800	-1.43167500
O	3.86864100	-0.37542700	-0.72095700
H	3.86363300	-0.53856300	0.23737000
H	3.53008700	0.52475300	-0.82181200
O	3.18136800	-0.71106800	2.00027300
H	3.61710500	-0.34532900	2.77415600
H	2.42781000	-0.13154900	1.80364400

72. TS23 G= -1474.770803 a.u.

S	0.81962900	-0.07234000	-0.74657600
O	-0.42448100	-0.13298700	-1.42898300
O	0.88159100	-0.36719900	0.64499700
O	1.98563900	0.35083500	-1.43172400
H	-1.18045100	2.87243600	0.06483200
H	-0.69811300	-2.12539900	0.09744500
N	-1.59159500	-1.93492400	0.55868600
H	-1.71590000	-0.87371900	0.58509000
C	-2.69073000	-2.55203800	-0.21772800
H	-2.57252100	-3.63375200	-0.20412600
H	-3.63461100	-2.25972100	0.23315200
H	-2.65085500	-2.16498100	-1.23140200
C	-1.52643500	-2.37769400	1.96803000
H	-2.45515600	-2.09234800	2.45603800
H	-1.39603200	-3.45671000	2.00908100
H	-0.69117700	-1.87175800	2.44495500

O	-3.42973100	0.33079800	-0.58520400
O	-0.31018700	3.28503100	0.42388700
N	0.61677800	2.38763500	0.21031600
N	-2.61531800	1.08683600	-0.07700200
O	-2.45275700	2.24849200	-0.50580300
O	1.67725200	2.67169800	0.67223200
O	-1.90280000	0.69495700	0.89167100
O	1.18150100	-2.37166000	-1.05611300
H	1.34204600	-2.58028500	-1.98231300
H	2.07134500	-2.36565700	-0.60191800
O	3.48689000	-2.02341600	0.14428900
H	3.56176400	-1.07881400	0.41559000
H	3.76092400	-2.53413200	0.91015900
O	3.81743600	0.56175700	0.98454700
H	4.47038800	1.01812700	0.44594200
H	3.02541400	1.11925800	0.92651500

73. $NH_2(CH_3)_2^+ \cdot HSO_4^- \cdot HNO_3 \cdot HNO_2 \cdot (H_2O)_2$ G= -1474.811926 a.u.

S	2.40001700	-0.60923100	-0.15740600
O	1.94206500	-1.85310600	-0.74014200
O	1.35877500	0.03620400	0.64983700
O	3.06939800	0.28103000	-1.08253000
H	-3.32484500	1.71697400	-0.05298400
H	0.21238400	-2.11310400	-0.27180700
N	-0.62114000	-2.42517400	0.25134000
H	-1.33120300	-1.65108700	0.25304000
C	-1.19379300	-3.62653200	-0.39555900
H	-0.46136200	-4.42973800	-0.35249600
H	-2.09903300	-3.90799500	0.13653000
H	-1.44492200	-3.38441100	-1.42278900
C	-0.20663800	-2.65363900	1.65438900
H	-1.07671000	-2.96401000	2.22807800
H	0.55768100	-3.42725100	1.67533300
H	0.19440400	-1.72356400	2.04663700
O	-3.09802500	-1.38960600	-1.31184100
O	-2.94686600	2.54625700	0.48605800
N	-1.67268400	2.44887100	0.37394400
N	-3.10071800	-0.42137400	-0.56761800
O	-3.74005500	0.61951500	-0.87361500
O	-1.08016800	3.31343700	0.98685400
O	-2.46076300	-0.43804100	0.51230700
O	3.48179800	-0.99564600	0.95466400
H	4.25696100	-1.37444900	0.51831400
H	3.27795800	3.46194700	-1.51055200
O	2.49317400	2.91102700	-1.44314500
H	1.76962100	2.83566900	-0.10421700
H	2.79027100	1.98025500	-1.51665100
O	1.32565000	2.56150800	0.77543000
H	0.36269200	2.91847500	0.84647900
H	1.32035800	1.53409000	0.77792700
